# The treatment with sGC stimulator improves survival of hypertensive rats in response to volume-overload induced by aorto-caval fistula

**DOI:** 10.1007/s00210-023-02561-y

**Published:** 2023-06-20

**Authors:** Olga Gawrys, Zuzana Husková, Petra Škaroupková, Zuzana Honetschlägerová, Zdeňka Vaňourková, Soňa Kikerlová, Vojtěch Melenovský, Barbara Szeiffová Bačová, Matúš Sykora, Miloš Táborský, Luděk Červenka

**Affiliations:** 1 Experimental Medicine Centre, Institute for Clinical and Experimental Medicine, Prague, Czech Republic; 2 Department of Cardiology, Institute for Clinical and Experimental Medicine, Prague, Czech Republic; 3 Centre of Experimental Medicine, Slovak Academy of Sciences, Institute for Heart Research, Bratislava, Slovakia; 4 Department of Internal Medicine I, Cardiology, University Hospital Olomouc and Palacký University, Olomouc, Czech Republic

**Keywords:** Heart failure, ACF, sGC stimulator, BAY41-8543, cGMP, Vericiguat

## Abstract

Heart failure (HF) has been declared as global pandemic and current therapies are still ineffective, especially in patients that develop concurrent cardio-renal syndrome. Considerable attention has been focused on the nitric oxide (NO)/soluble guanylyl cyclase (sGC)/cyclic guanosine monophosphate (cGMP) pathway. In the current study, we aimed to investigate the effectiveness of sGC stimulator (BAY41-8543) with the same mode of action as vericiguat, for the treatment of heart failure (HF) with cardio-renal syndrome. As a model, we chose heterozygous Ren-2 transgenic rats (TGR), with high-output heart failure, induced by aorto-caval fistula (ACF). The rats were subjected into three experimental protocols to evaluate short-term effects of the treatment, impact on blood pressure, and finally the long-term survival lasting 210 days. As control groups, we used hypertensive sham TGR and normotensive sham HanSD rats. We have shown that the sGC stimulator effectively increased the survival of rats with HF in comparison to untreated animals. After 60 days of sGC stimulator treatment, the survival was still 50% compared to 8% in the untreated rats. One-week treatment with sGC stimulator increased the excretion of cGMP in ACF TGR (109 ± 28 nnmol/12 h), but the ACE inhibitor decreased it (-63 ± 21 nnmol/12 h). Moreover, sGC stimulator caused a decrease in SBP, but this effect was only temporary (day 0: 117 ± 3; day 2: 108 ± 1; day 14: 124 ± 2 mmHg). These results support the concept that sGC stimulators might represent a valuable class of drugs to battle heart failure especially with cardio-renal syndrome, but further studies are necessary.

**Supplementary information:**

The online version contains supplementary material available at 10.1007/s00210-023-02561-y.

## Introduction

Heart failure (HF) represents a major health and socioeconomic burden with considerable morbidity and mortality. It is estimated that it affects around 23 million people globally (Murphy et al. 2020). HF can be categorised into three main groups based on the ejection fraction (EF), i.e., heart failure with reduced EF (HFrEF), heart failure with mid-range or mildly reduced EF (HFmrEF), and heart failure with preserved ejection fraction (HFpEF) (Simmonds et al. 2020). Over the recent decades a significant improvement has been made in the management and treatment of HF. Current guideline-directed medical therapy (GDMT) includes combination of β-blockers, angiotensin-converting enzyme inhibitors (ACEi), angiotensin receptor–neprilysin inhibitors (ARNI), angiotensin receptor blocker (ARB) with an addition of a mineralocorticoid receptor antagonists, diuretics and more recently sodium-glucose cotransporter 2 (SGLT2) inhibitors (McDonagh et al. 2021; Heidenreich et al. 2022). The prognosis and life expectancy is especially dreadful for patients that develop concurrent impairment of renal hemodynamic and sodium excretory function, so called cardio-renal syndrome (Rangaswami et al. 2019; McCullough et al. 2022). It is now commonly acknowledged that the reciprocal interaction between cardiac and renal function plays critical role in the progression of HF (Mullens et al. 2017; Ciccarelli et al. 2021; McCullough et al. 2022). Current GDMT are still ineffective in the treatment of HF, especially with cardio-renal syndrome, and the morbidity and mortality still remain very high, with a 5-year survival rate of 25% after first hospitalization (Berliner et al. 2020; Murphy et al. 2020). Hence new treatment strategies are still urgently needed to improve the morbidity and mortality.

Lately particular attention has been focused on the nitric oxide (NO)/soluble guanylyl cyclase (sGC)/cyclic guanosine monophosphate (cGMP) pathway. In the classical cascade NO binds to sGC, which leads to a conversion of guanosine triphosphate (GTP) into cGMP (Singh et al. 2018). In turn, this secondary messenger, cGMP, acts on various effector molecules including protein kinases (PK) or phosphodiesterases (PDE), initiating a number of downstream effects, such as vasodilation and myocardial relaxation. It was proved to exhibit anti-inflammatory, anti-proliferatory, anti-fibrosis and renal protective properties, making this pathway one of the most essential signalling cascades within the cardiovascular and renal systems. Its disruption can lead to development of serious disorders or progression of already existing diseases, such as heart failure, pulmonary hypertension or chronic kidney disease (Farah et al. 2018; Sandner et al. 2021a; Xia et al. 2022).

So far several drug classes targeting NO-sGC-cGMP pathway have been developed, such as NO donors (e.g. nitroglycerine) or PDE5 inhibitors (e.g., sildenafil) (Roberto et al. 2012) and more recently sGC stimulators and sGC activators were introduced (Cordwin et al. 2021). The sGC activators (e.g. cinaciguat) activate both the oxidized and heme-free sGC, while sGC stimulators increase cGMP production independently of NO by activating the reduced heme moiety (Cordwin et al. 2021). sGC stimulators, such as riociguat and vericiguat, exhibit also synergistic activity with NO, because they sensitize sGC to low levels of endogenous NO by stabilizing NO–sGC binding (Stasch et al. 2001; Liu et al. 2021). Vericiguat was recently approved for the treatment of heart failure with reduced ejection fraction based on positive outcome of a large clinical trial in patients with HFrEF (Armstrong et al. 2020). However, the exact mode of action of sGC stimulators and how these beneficial effects in HF are mediated are not fully understood yet and particularly it is unknown if it will be effective in cardio-renal syndrome.

In the current work, we therefore, treated hypertensive, heterozygous Ren-2 transgenic rats (TGR), with high-output heart failure, induced by creating an aorto-caval fistula (ACF) with the sGC stimulator (BAY41-8543), which exhibits the same mode of action as riociguat and vericiguat (Sandner et al. 2021b). ACF TGR model is a well-established model of volume-overload heart failure and it is routinely used in our laboratory for many years (Abassi et al. 2011; Honetschlägerová et al. 2021; Kala et al. 2021, 2023), because it represents a model of HF accompanied with development of cardio-renal syndrome. In addition, we also investigated an optimized dose of an ACE inhibitor (trandolapril) as the standard therapy for HF as positive control.

## Methods

### Animals

The studies were performed in accordance with guidelines and practices established by the Animal Care and Use Committee of the Institute for Clinical and Experimental Medicine (Prague) approved by the Ministry of Health of the Czech Republic (decision number MZDR 12482/2021–5/OVZ), which accords with the European Union Directive 63/2010 and ARRIVE guidelines (Animal Research: Reporting of In Vivo Experiments).

All animals used in the present study were bred at the Center of Experimental Medicine of this Institute (IKEM), from stock animals supplied by the Max Delbrück Center for Molecular Medicine (Berlin, Germany), which is accredited by the Czech Association for Accreditation of Laboratory Animal Care. Heterozygous TGR [transgenic rats, strain name TGR(mRen2)27] harboring the mouse Ren-2 renin gene have been recently generated as a model for the study of primary hypertension. They were generated by breeding male homozygous TGR with female homozygous Hannover-Sprague Dawley (HanSD) rats. Age-matched HanSD rats served as transgene-negative normotensive controls. The animals were kept on a 12-h/12-h light/dark cycle and had free access to tap water throughout the whole observation. Male TGR rats at the initial age of 8 weeks were used for experiments. At this age TGR are already in the sustained phase of hypertension with systolic blood pressure (SBP) comparable with hypertensive patients (SBP around 180 mmHg) and with substantial activation of endogenous renin angiotensin system (RAAS), as demonstrated in previous studies including ours. HanSD and TGR rats were randomly assigned to experimental groups to make sure that the animals from a single litter does not prevail in any group.

### Heart failure model and exclusion criteria

Eight-weeks-old male TGR rats were anesthetized with an intraperitoneal injection of ketamine/midazolam mixture (Calypsol, Gedeon Richter, Hungary, 160 mg/kg and Dormicum, Roche, France, 160 mg/kg). Chronic HF due to volume overload was then induced by creating an aorto-caval fistula (ACF) using a needle technique. This procedure is routinely performed in our laboratory and detailed description was reported repeatedly in our previous studies (Honetschlägerová et al. 2021; Kala et al. 2021, 2023). Sham-operated rats underwent an identical procedure, but without creating ACF. The animals in which the ACF procedure was not successful (based on visual verification of vena cava inferior in the end of each observation) were excluded from the experiment.

### Detailed experimental design

**Fig. 1:**
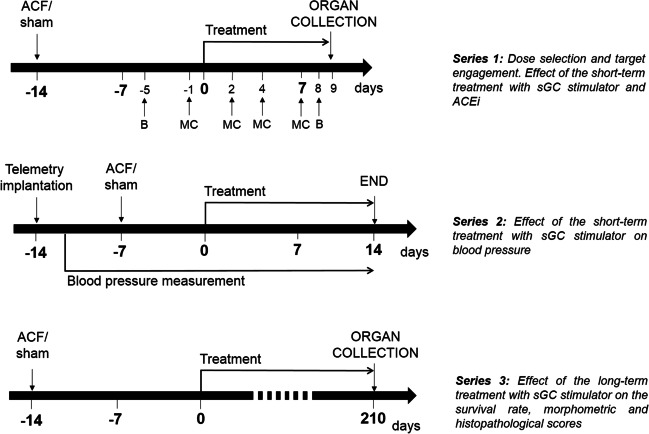
The experimental design of the whole study depicting the time sequence and experimental manoeuvres; MC – metabolic cage with 12 h urine collection; B – blood collection from the tail vein

Detailed experimental design of all 3 series is presented on Fig. 1.

#### Series 1: Dose selection and target engagement. Effect of the short-term treatment with sGC stimulator and ACEi

Two weeks before the start of the treatment rats underwent sham-operation or ACF creation procedure. After two weeks (week labelled 0) and after exclusion of acute death cases, the rats were randomly divided into the following experimental groups and selected treatment regimens were applied for one week. On days -1, 2, 4, and 7 rats were placed in metabolic cages for 12 h urine collection. Also, blood was collected on days -5 and 8. After a week of the treatment, all rats were decapitated and organs were weighted and collected for further biochemical evaluation.cGMP was measured in the urine and in the renal tissue. Noradrenaline and angiotensin II were measured in the plasma and the kidneys (collected after decapitation). Albuminuria, natriuresis, and daily excretion rate of nitric oxide (NO) metabolites (NOx: nitrate and nitrite; indirect marker of NO production) were evaluated. Additionally, to confirm that the selected dose was effective we have measured the concentration of BAY41-8543 in plasma.

#### Series 2: Effect of the treatment with sGC stimulator and ACEi on blood pressure (BP)

Ten days before the ACF creation, telemetry probes were implanted into femoral artery of TGR rats (HanSD rats were omitted in this part of the study) under ketamine/midazolam anaesthesia (as above). HD-S10 radiotelemetric probes (Data Science International, St. Paul, Minnesota, USA) were used for direct BP measurements as described previously (Sporková et al. 2014; Husková et al. 2016).

After 10-day period of recovery, rats underwent either sham operation or ACF creation as described above. A week after the appropriate treatment was initiated and rats were monitored for two weeks.

#### Series 3: Effects of the long-term treatment with sGC stimulator and ACEi on the survival rate, morphometric and histopathological scores

After confirmation that the selected dose of sGC stimulator is effective, the long-term protocol was performed. All rats underwent the same ACF creation or sham operation as described above in Series 1. After 2 weeks (week labelled 0), after exclusion of acute death cases, the rats were randomly divided into the following experimental groups and the follow-up period of 210 days was performed. Because of the severity of the ACF procedure and high mortality (especially in TGR rats) high initial n values were used in these groups (*n* = 30). In sham operated animals and HanSD rats the initial number of animals was 10 (calculated by statistical power analysis method).

After 210 days of the observation, the surviving animals were decapitated and organs were weighted and collected for morphometric and histopathological analysis (left and right ventricle of the heart, kidney).

### Experimental groups

1. Sham-operated HanSD rats treated with placebo (sham HanSD)
2. Sham-operated HanSD rats treated with BAY41-8543 (sham HanSD + sGCstim)
3. Sham-operated TGR treated with placebo (sham TGR)
4. Sham-operated TGR treated with BAY41-8543 (sham TGR + sGCstim)
5. ACF TGR treated with placebo (ACF TGR)
6. ACF TGR treated with BAY41-8543 (ACF TGR + sGCstim)
7. ACF TGR treated with trandolapril (ACF TGR + ACEi)
8. ACF TGR treated with trandolapril and BAY41-8543 (ACF TGR + ACEi + sGCstim)

The numbers of rats used in each series is described in the appropriate result section.

### Analytical procedures and chemicals

The sGC stimulator BAY41-8543 (2-[1-[(2-fluorophenyl)methyl]-1H-pyrazolo[3,4-b]pyridin-3-yl]-5(4-morpholinyl) -4,6-pyrimidinediamine) was kindly provided by Bayer AG, Pharmaceuticals and is a typical member of the sGC stimulator drug class (Stasch et al. 2002a, b). Standard pellet diet containing BAY41-8543 in the dose of 3 mg kg^−1^ day^−1^ was prepared (Albert Weber, Prague, Czech Republic). Nutrients (content in 1 kg): NL 200 g; Fiber 48 g; Fat 30 g; Vitamin A 24.000 IU; Vitamin D3 2.000 IU; Copper (Cu) 30 mg; NaCl content in the final mix 0.4%. The dose of BAY41-8543 was selected based on the previous study (Stasch et al. 2002b; Sandner et al. 2021b) and current research.

As an ACE inhibitor (ACEi) we used trandolapril (Gopten; Abbott, Prague, Czech Republic), which was administered in drinking water. For the best effectiveness and safety, we implemented a titration protocol, which was previously developed and validated in our laboratory. During the first week of administration, the animals received an increasing dose (changed every two days) starting from 0.5 mg/L up to 2 mg/L, which corresponds to a final dose of 0.25 mg kg^−1^ day^−1^. In our previous studies and here we demonstrated that this titration regimen and selected doses of trandolapril, provided maximal blockade of the renin angiotensin system (RAAS) and were well tolerated both by rats with ACF-induced heart failure and by sham-operated animals.

### SDS-PAGE and Western blotting

According to our previous studies (Szeiffová Bačova et al. 2016; Sykora et al. 2023), approximately 100 mg of frozen left ventricular heart tissue was homogenized in lysis buffer [50 mmol/L Tris–HCl, 250 sucrose, 1.0 mmol/L EGTA, 1.0 mmol/L dithiothreitol, 1.0 mmol/L phenylmethylsulfonyl fluoride and 0.5 sodium orthovanadate (pH 7.4)] and mixed with Laemmli sample buffer. Loading samples were separated in 10% SDS-PAGE (Mini-Protean TetraCell, Bio-Rad, Hercules, CA, USA) and transferred to a nitrocellulose membrane (0.2 µm pore size, Advantec, Tokyo, Japan). Membranes were subsequently incubated for 4 h with 5% low-fat milk, overnight with primary antibodies and for 1 h with a horseradish peroxidase-linked secondary antibody (Table 1). Between individual steps were membranes washed in TBS-T. Protein were visualized by enhanced chemiluminescence method and quantitated by densitometric analysis using Carestream Molecular Imaging Software (version 5.0, Carestream Health, New Haven, CT, USA).

**Table 1:** Antibodies used for Western blot analysis and for immunofluorescence methods

Antibody	Dilution	Host	Type	Supplier/# Catalogue
anti-Cx43	1:5000	Rabbit	Polyclonal	Sigma-Aldrich, St.Louis, MO, USA, #C6219
anti-phospho-ser368-Cx43	1:1000	Rabbit	Polyclonal	Santa Cruz Biotechnology, Dallas, TX, USA, #sc-101660
anti-PKC-epsilon	1:2000	Rabbit	Polyclonal	Santa Cruz Biotechnology, Dallas, TX, USA, #sc-214
anti-GAPDH	1:1000	Rabbit	Polyclonal	Santa Cruz Biotechnology, Dallas, TX, USA#sc-25778
Anti-Rabbit	1:2000	-	-	Cell Signaling Technology, Danvers, MA, USA, #7074S
anti-Cx43	1:500	Mouse	Monoclonal	CHEMICON International,CA, USA, #MAB 3068
Anti-Mouse, FITC	1:500	Goat	Polyclonal	Jackson Immuno Research Labs, West Grove, Pennsylvania, USA, #115–095-062

### Immunofluorescence detection of Cx43 and quantitative image analysis

Immunodetection of Cx43 distribution was performed as described previously (Benova et al. 2013). Briefly, 10 µm thick left ventricular cryosections were washed in phosphate buffer saline (PBS), fixed in ice-cold methanol, permeabilized in 0.3% Triton X-100 in PBS, and blocked with the solution of 1% bovine serum albumin in PBS. Tissue sections were incubated overnight with primary antibody followed by 2-h incubation with secondary antibody (Table 1). Between individual steps were membranes washed in PBS. Finally, tissues sections were mounted in the Fluoromount-G™ Mounting Medium (00–4958-02, Invitrogen™, Massachusetts, USA) and analysed by Zeiss Apotome 2 microscope (Carl Zeiss, Jena, Germany). Approximately ten randomly acquired images from every tissue were captured and analyzed. Immunofluorescence signals were analysed and defined as a number of pixels with the protein signal intensity exceeding a threshold of 30 on the 0–255 Gy scale. The total number of Cx43 positive pixels was expressed as a total integral optical density per area (IOD) (Image-Pro Plus) (Sykora et al. 2019).

### Evaluation of hydroxyproline content

Measurement of hydroxyproline is a useful method to determine collagen content in the samples. Briefly, left ventricle tissue was dried, hydrolyzed in 6 M HCl and oxidated by chloramine T in the acetate-citrate buffer (pH 6.0). This reaction was stopped by pipetting Ehrlich’s reagent solution. Final concentration of hydroxyproline was subsequently measured spectrophotometrically at 550 nm and expressed in mg per total weight of the left ventricle (Pelouch et al. 1993; Reddy and Enwemeka 1996).

### Histology and enzyme histochemistry of myocardial tissue

According to Lojda (Lojda and Gutmann 1976) with modifications (Andelova et al. 2022), 10 µm thick left ventricular myocardial tissue cryosections were used for histological and histochemical techniques.

For myocardial structural changes characterization, a conventional histological haematoxylin–eosin staining was used. Briefly, dried cryosection were fixed in 4% buffered formaldehyde, stained with hematoxylin–eosin solutions, poured with Canada balsam and covered with a coverslip. To stain collagen deposition, Van Gieson technique was performed. Heart tissue sections were also fixed within 4% buffered formaldehyde, incubated in a mixture of saturated picric acid and 1% aqueous acid fuchsine, and subsequently covered by Canada balsam and coverslips.

For measurement of capillary endothelium-related alkaline phosphatase AP (E.C.3.1.3.1), cryosections were incubated in the mixture of solution (2,7 mM Naphthol AS-MX phosphate; 4,8 mM Fast blue BB; 5% dimethylformamide; 0,1 M Tris(hydroxymethyl)aminomethane). Subsequently, the slices were rinsed in distilled water and placed in a 2% copper sulfate solution and finally covered by gelatine and coverslips. To detect functional arteriolar and venular capillary network, dipeptidyl peptidase-4 (DPP4, E.C.3.4.15.4) was examined. Cryosections were incubated in solution (1.2 mM L-Leucine 4-methoxy-β-naptylamide hydrochloride; 5% dimethylformamide; 2.4 mM Fast blue BB; 0.1 M Na2 HPO4 × 2H2O; 1 M KH2PO4), poured with gelatine and covered with a coverslip.

Staining areas were observed and captured by light microscope (Zeiss Apotome 2 microscope Carl Zeiss, Jena, Germany). For quantitative analysis, randomly selected areas of positive signal from every tissue were analysed. A positive signal was expressed as a proportion of the total tissue area (Andelova et al. 2022).

### Evaluation of glomerulosclerosis index (GSI) and tubulointerstitial injury (TSI)

The kidneys were fixed in 4% formaldehyde, dehydrated and embedded in paraffin. The sections stained with hematoxylin–eosin and PAS (periodic acid, for Schiff reaction) were examined and evaluated in a blind-test fashion. The calculation of glomerulosclerosis index (GSI) and kidney cortical tubulointerstitial injury (TSI) was described in detail in previous studies (Nakano et al. 2008; Gawrys et al. 2018; Honetschlagerová et al. 2021; Kala et al. 2023). The maximum score for GSI is 4 and for TSI is 3. Described methods are commonly employed in our laboratory for many years and standardly used for evaluating the degree of kidney damage in almost all our studies (Kujal et al. 2014; Sedláková et al. 2017; Honetschlagerová et al. 2021).

Plasma and tissue angiotensin II (ANG II) concentrations were measured by a competitive radioimmunoassay, using the commercially available RIA kit (ED29051, IBL Int., Hamburg, Germany). Plasma creatinine was measured by FUJI DRI-CHEM analyzer using appropriate slides for creatinine CRE-P III (FUJIFILM Corp., Tokyo, Japan). Urine creatinine was determined using Liquick Cor-CREATININE kit that is based on modified Jaffe’s method, without deproteinization (PZ CORMAY S.A., Poland). Nitrate/nitrite levels were measured by a colorimetric assay (780,001, Cayman Chemical, Ann Arbor, MI, USA).

Commercially available ELISA kits were used to measure: renal nitrotyrosine (ab113848; Abcam, Cambridge, UK); renal cGMP (ADI-900–013; Enzo, Farmingdale, NY, USA) and plasma cGMP (581,021; Cayman Chem., Ann Arbor, MI, USA); plasma and renal noradrenalin (RE59261; IBL Int., Hamburg, Germany); urine 8-isoprostane (516,351; Cayman Chem., Ann Arbor, MI, USA). Sodium and potassium in plasma and urine were measured by BWB-XP flame photometer (BWB Technologies Ltd., Berkshire, UK). Detailed protocols of plasma and tissue preparation are described in our previous studies (Husková et al. 2006, 2007, 2016; Červenka et al. 2015a; Kratky et al. 2018; Gawrys et al. 2020).

### Data and statistical analysis

All values are expressed as means ± SEM. Graph-Pad Prism software (Graph Pad Software, San Diego, California, USA) was used for statistical analysis of the data. Comparison of survival curves was performed by log-rank (Mantel-Cox) test. Multiple-group comparisons were performed by multiple *t* test, Wilcoxon´s signed-rank test, one-way or two-way analysis of variance followed by recommended post hoc test as appropriate. Values exceeding the 95% probability limits (*P* < 0.05) were considered statistically significant. The significance levels are indicated on figures with asterisks: *P* > 0.05 (NS); *P ≤ 0.05; **P ≤ 0.01; ***P ≤ 0.001; ****P ≤ 0.0001. The data and statistical analysis comply with the recommendations on experimental design and analysis in pharmacology (Curtis et al. 2018).

## Results

### Series 1: Dose selection and target engagement. Effect of the short-term treatment with sGC stimulator and ACEi (Figs. 2 and 3; Table 2)

**Fig. 2:**
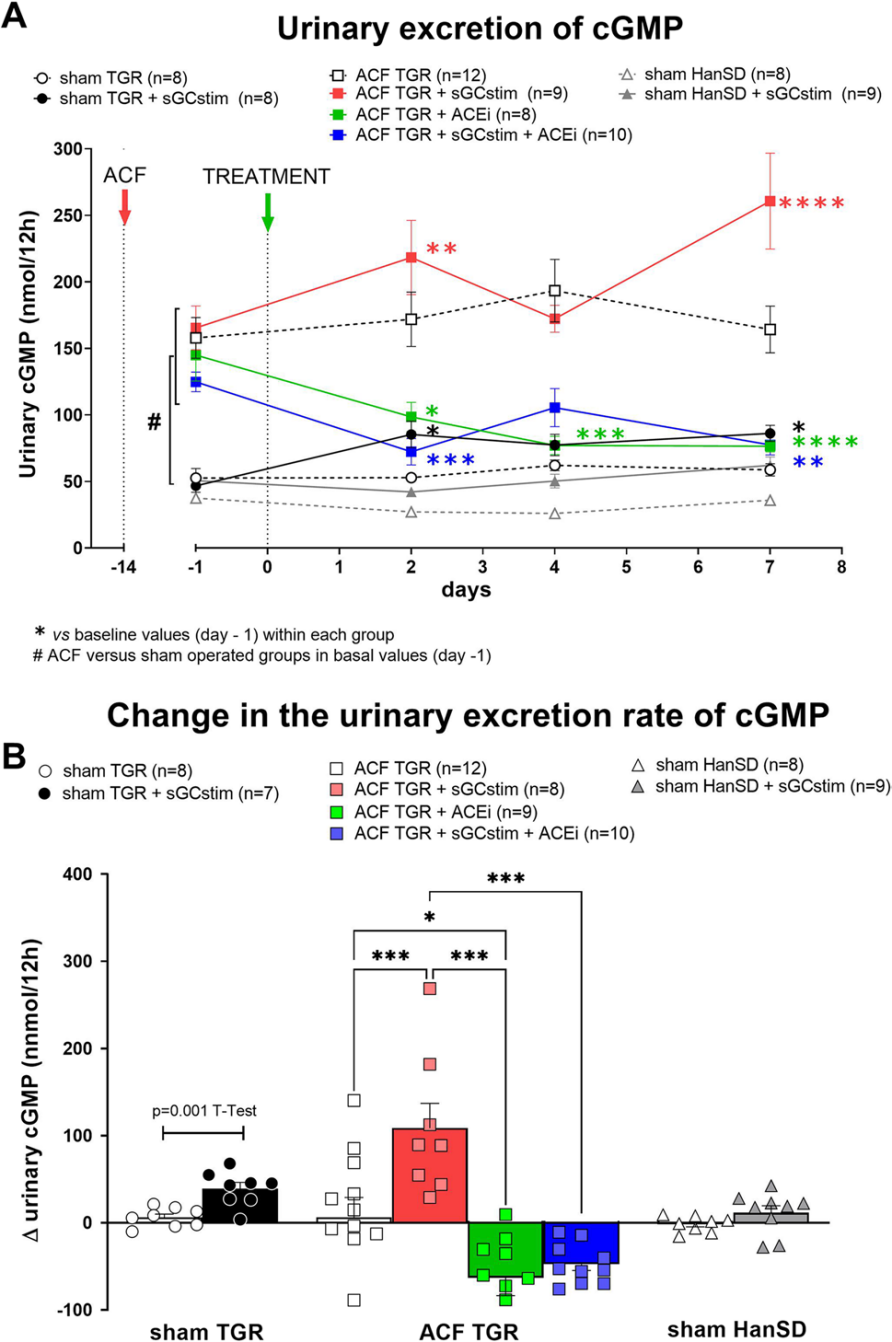
Urinary excretion of cGMP **A**: throughout the whole observation and **B**: the difference (Δ) between basal values (day -1) and the end (day 7) in heterozygous Ren-2 transgenic rats (TGR) rats with aorto-caval fistula (ACF) or without (sham) and normotensive sham HanSD rats treated with sGC stimulator (BAY41-8543), or with angiotensin-converting enzyme inhibitor (ACEi), alone or combined; *P* > 0.05 (NS); * *P* ≤ 0.05; ** *P* ≤ 0.01; *** *P* ≤ 0.001; **** *P* ≤ 0.0001; by 2-way ANOVA with Dunnett's (within each group) and Tukey’s (between groups) multiple comparisons tests; # *P* ≤ 0.0001 basal values after ACF *versus* sham operation (day-1) by unpaired *T*-test

The first series of experiments was aimed to evaluate the short-term effects of treatment with sGC stimulator to support dose selection and demonstrate target engagement. As a marker of target engagement for the sGC stimulator treatment, cGMP excretion in urine was measured. As depicted in Fig. 2 the excretion of cGMP increased in all groups treated with sGC stimulator (BAY41-8543). The treatment with ACEi decreased the cGMP levels in urine which could not be compensated by sGC stimulator treatment. Interestingly, before the treatment the basal values of urinary cGMP were significantly higher in all groups with heart failure after ACF creation (148.5 ± 7.6 *versus* 49.6 ± 4.2 nmol/12 h in sham operated TGR, # *P* < 0.0001 unpaired *T*-Test; Fig. 2A).

**Fig. 3:**
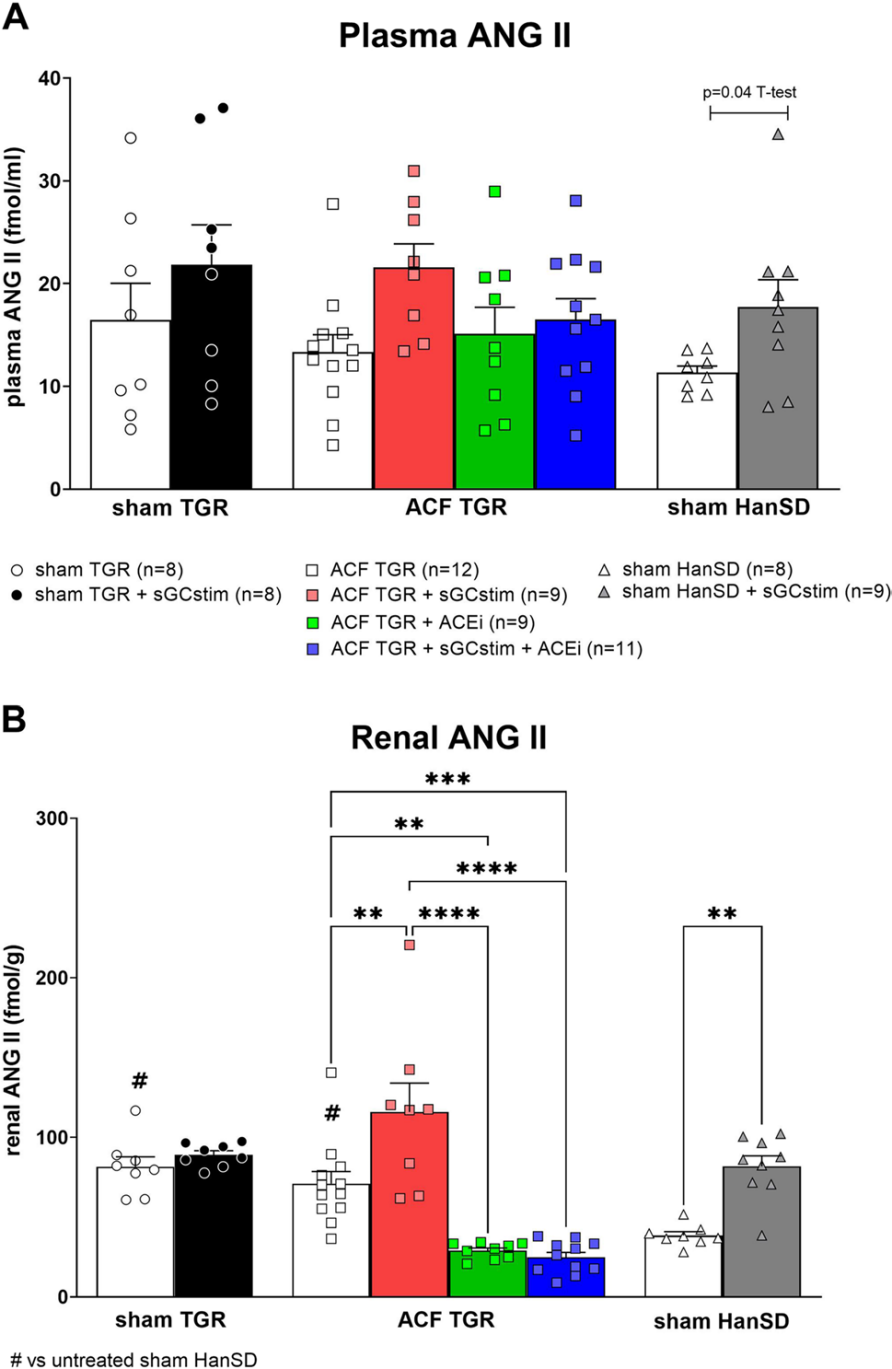
Angiotensin II (ANG II) **A**: in plasma and **B**: in renal tissue in heterozygous Ren-2 transgenic rats (TGR) rats with aorto-caval fistula (ACF) or without (sham) and normotensive sham HanSD rats after 7 day treatment with sGC stimulator (BAY41-8543), or with angiotensin-converting enzyme inhibitor (ACEi), alone or combined. *P* > 0.05 (NS); * *P* ≤ 0.05; ** *P* ≤ 0.01; *** *P* ≤ 0.001; **** *P* ≤ 0.0001 by one-way ANOVA with Tukey’s multiple comparisons tests; # *p* < 0.05 *versus* sham HanSD by one way ANOVA with Tukey’s multiple comparisons tests

**Table 2:** Body weight (BW), plasma and urine parameters collected from heterozygous Ren-2 transgenic rats (TGR) rats with aorto-caval fistula (ACF) or without (sham) and normotensive sham HanSD rats treated for one week with sGC stimulator (BAY41-8543), or with angiotensin-converting enzyme inhibitor (ACEi), alone or combined (n = 8–13 in each group);

	sham TGR	sham TGR + sGCstim	ACF TGR	ACF TGR + sGCstim	ACF TGR + ACEi	ACF TGR + sGCstim + ACEi	sham HanSD	sham HanSD + sGCstim
	a	b	c	d	e	f	g	h
BW (g)	449 ± 13	441 ± 6	408 ± 5	429 ± 10	415 ± 9	425 ± 16	455 ± 9	470 ± 10
UNa V (mmol/12 h)	1.7 ± 0.1	2.0 ± 0.2	0.7 ± 0.1	0.6 ± 0.1	1.0 ± 0.1	1.2 ± 0.1 ^c,d^	1.0 ± 0.1	0.6 ± 0.1
UK V (mmol/12 h)	2.4 ± 0.2	2.0 ± 0.1	1.8 ± 0.1	1.7 ± 0.1	1.9 ± 0.1	2.1 ± 0.1	1.6 ± 0.1	1.6 ± 0.1
UNO_x_ (μmol/12 h)	5.2 ± 0.1	4.2 ± 0.4 ^e,g^,*	5.2 ± 0.5	4.3 ± 0.4 ^e,g^, #	7.4 ± 0.6 h	4.6 ± 0.6 ^e,g^	7.3 ± 0.4	3.4 ± 0.4 ^c,g^
U 8-isoprostane (ng/12 h)	8.5 ± 1.2	12.7 ± 1.1	15.8 ± 1.8 ^a^	15.3 ± 1.3	12.5 ± 1.7	12.0 ± 1.8	7.8 ± 0.6 ^c,d^	9.9 ± 1.2
Plasma Na (mmol/L)	137 ± 4	143 ± 5	139 ± 3	137 ± 4	136 ± 2	131 ± 2	134 ± 3	132 ± 2
Plasma K (mmol/L)	4.4 ± 0.2	4.3 ± 0.1	4.8 ± 0.1	4.5 ± 0.1	5.1 ± 0.2	4.6 ± 0.1	4.4 ± 0.1	4.0 ± 0.1
Plasma Cr (µmol/L)	15 ± 1	14 ± 1	18 ± 1	16 ± 1	17 ± 1	21 ± 2	18 ± 2	13 ± 1
Plasma NE (ng/ml)	2005 ± 420	3586 ± 856	2989 ± 192	2095 ± 478	3174 ± 1110	2899 ± 740	1560 ± 579	631 ± 93
Renal NE (ng/g prot.)	211 ± 21	161 ± 12 ^d^	278 ± 20	264 ± 39	286 ± 59	338 ± 51	293 ± 22	239 ± 17
Renal Nitrotyrosine (ng/mg prot.)	26.5 ± 2.3	18.5 ± 1.1	19.7 ± 1.6	17.1 ± 2.4	18.5 ± 2.7	15.1 ± 2.0 ^a^	20.7 ± 3.5	16.3 ± 2.0
Plasma BAY41-8543 (µg/L)	-	29.7 ± 4.5	-	30.8 ± 5.6	-	19.7 ± 2.0	-	26.7 ± 3.4

*BW* body weight; *UNa V* urinary sodium excretion; *UK V* urinary potassium excretion; *UNO*_*x*_ urinary excretion of nitric oxide metabolites (nitrate/nitrite); *Cr* creatinine; *NE* noradrenaline; Superscript letter given next to the value (a–g) indicate statistically significant difference versus group indicated in the second row of the table (under the group’s name) by one way ANOVA with Tukey's multiple comparisons test

^*^ *p* < 0.05 sham TGR + sGCstim. vs untreated sham TGR by unpaired *t* test; # *p* < 0.05 ACF TGR + sGCstim. vs untreated ACF TGR by unpaired *t* test

Body weight, plasma (sodium, potassium, creatinine, noradrenaline), urine parameters (sodium, potassium, nitric oxide metabolites and 8-isoprostane excretion) and renal levels of noradrenaline and nitrotyrosine collected after one week treatment are presented in Table 2. Urinary excretion of nitric oxide metabolites (NOx: nitrate and nitrite) was measured as an indirect marker of NO production. In all the groups which received sGC stimulator, there was a significant or tendency to decrease the NOx excretion, especially pronounced in sham TGR and sham HanSD rats. The treatment with ACEi increased the excretion of NOx, but after combined therapy with sGC stimulator it was diminished.

Plasma and renal levels of ANG II are presented on Fig. 3 (panel A and B, respectively). Renal ANG II were significantly higher in both sham TGR and ACF TGR in comparison to control sham HanSD rats (81.6 ± 6.2 and 71.0 ± 7.6 *versus* 38.5 ± 2.4 fmol/g, respectively; # *p* < 0.05 one way ANOVA; Fig. 3). The treatment with sGC stimulator significantly increased ANG II in sham HanSD rats, both plasma (17.7 ± 2.6 *versus* 11.3 ± 0.6 fmol/ml in untreated sham HanSD; *p* < 0.05 one way ANOVA) and kidney (81.8 ± 6.6 *versus* 38.5 ± 2.4 fmol/g in untreated sham HanSD; *p* < 0.05 one way ANOVA); and in ACF TGR rats in the kidney (115.9 ± 18.1 *versus* 71.0 ± 7.6 fmol/g in untreated TGR rats; *p* < 0.05 one way ANOVA). Also some increasing tendency was observed in plasma ANG II after sGC stimulator administration to sham TGR (21.8 ± 3.9 *versus* 16.4 ± 3.6 fmol/ml in untreated sham TGR; NS) and to ACF TGR (21.6 ± 2.3 *versus* 13.3 ± 1.7 fmol/ml in untreated ACF TGR; *p* < 0.05 by unpaired *T*-test). Treatment with ACEi inhibitor decreased the renal level of ANG II, alone and also when combined with sGC stimulator.

To confirm that the selected dose is effective and the rats eat the proper amount of the food mixed with the sGC stimulator, we monitored the food intake and we measured the concentration of BAY41-8543 in plasma in the end of the experiment. There were no differences between groups in the food intake and the plasma levels of sGC stimulator were appropriately elevated in all treated groups (Table 2).

### Series 2: Effect of the treatment with sGC stimulator and ACEi on blood pressure (Figs. 4 and 5)

**Fig. 4:**
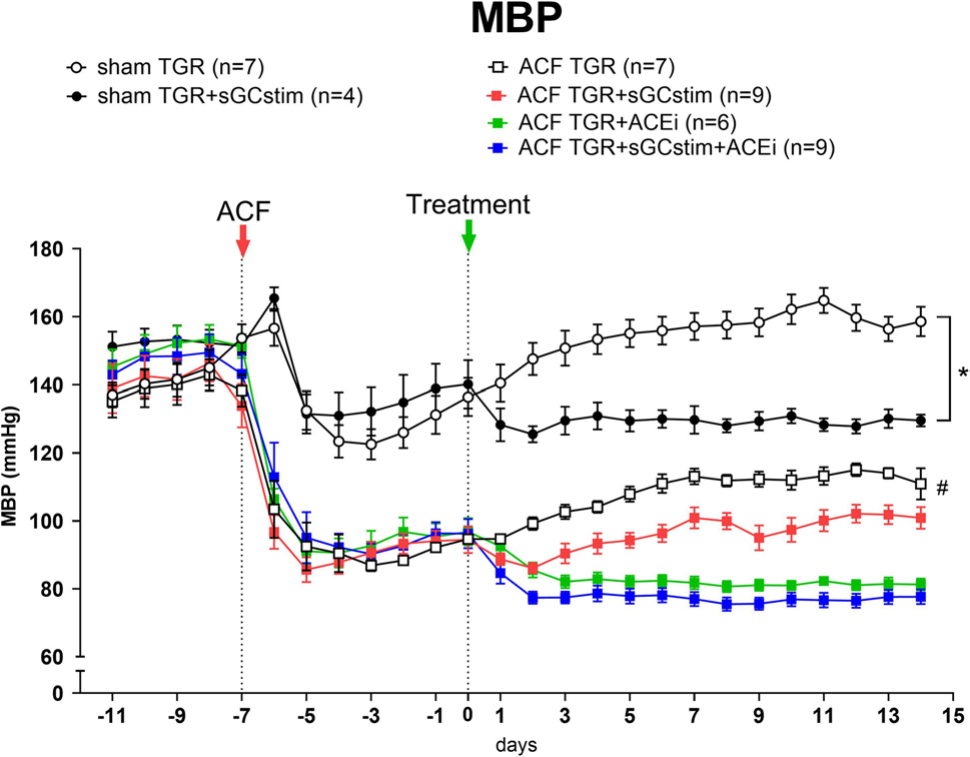
Time course of mean arterial blood pressure (MBP) throughout the whole observation in heterozygous Ren-2 transgenic rats (TGR) rats with aorto-caval fistula (ACF) or without (sham) treated with sGC stimulator (BAY41-8543), or with angiotensin-converting enzyme inhibitor (ACEi), alone or combined. * *P* < 0.05 by 2-way ANOVA with Bonferroni's multiple comparisons test; # *P* < 0.05 *versus* all other groups 2-way ANOVA with Tukey’s multiple comparisons tests

**Fig. 5:**
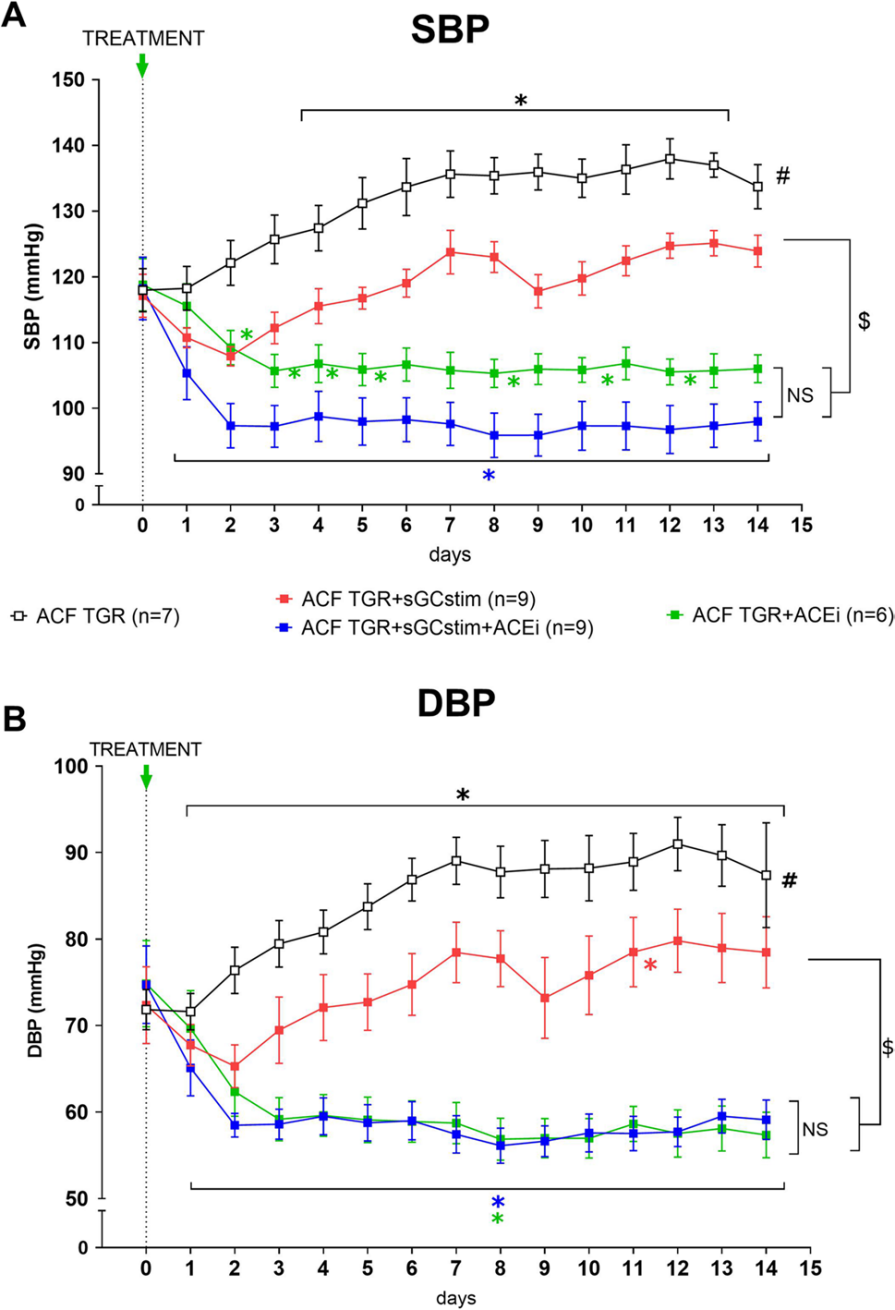
Time course of (**A**) systolic (SBP) and (**B**) diastolic (DBP) blood pressure throughout 2-week observation in heterozygous Ren-2 transgenic rats (TGR) rats with aorto-caval fistula (ACF) treated with sGC stimulator (BAY41-8543), or with angiotensin-converting enzyme inhibitor (ACEi), alone or combined. * *P* < 0.05 *versus* baseline values on day 0 within each group; # values different between all other groups; $ *P* < 0.05 values different between BAY41-8543 and both ACEi treated groups of ACF TGR by 2-way ANOVA with Dunnet’s (within each group) and Tukey’s (between groups) multiple comparisons tests. The BP values before the treatment and sham-operated groups are omitted for clarity

Blood pressure (BP) was measured by telemetry starting four days before ACF creation. The creation of ACF per se caused a significant decrease in mean arterial blood pressure (MBP, Fig. 4), which is a common phenomenon observed in this model (-52 ± 2 mmHg in all ACF operated groups) (Červenka et al. 2015b). In sham operated animals, the temporary decrease in blood pressure was also observed, probably as a result of anaesthesia and fluid loss during the operation.

The treatment with sGC stimulator in sham operated TGR animals caused a significant MBP decrease in comparison to untreated sham TGR (day 0: 140 ± 7 *versus* 130 ± 2 mmHg after 14 days of treatment). In addition, all applied treatment regimens decreased blood pressure in comparison to untreated ACF TGR (Fig. 4) including the treatment with sGC stimulator; however, the decrease in this groups was only transient (day 0: 94 ± 4 *versus* 86 ± 2 mmHg after 2 days of treatment; *p* = 0.03 paired *T*-test). The course of MBP changes was different between sGC stimulator group and ACEi treated groups, but there were no significant differences between both ACEi treated groups (ACF TGR + ACEi *versus* ACF TGR + sGCstim + ACEi, NS).

In order to better illustrate and evaluate the magnitude of antihypertensive potency of each administered drug, only ACF groups are presented on Fig. 5 (without sham-operated animals) and the values before the treatment are omitted (before and after ACF creation). The comparison of BP changes within each treated group (compared to baseline values obtained before the implementation of treatments) revealed that the most potent in lowering systolic blood pressure was the combined treatment with ACEi and sGC stimulator (SBP on day 0: 118 ± 5 *versus* 98 ± 3 mmHg on day 14). In addition, ACEi given alone was clearly antihypertensive, however the statistical analysis did not reveal such strong effect on SBP (SBP on day 0: 119 ± 4 *versus* 106 ± 2 mmHg on day 14). However, when DBP values were analyzed both ACEi treated groups (alone and combined with sGC stimulator) were similarly effective.sGC stimulator caused a decrease in BP (both SBP and DBP) especially pronounced in the beginning, but this effect was only transient and less significant than for other treatment regimens (SBP day 0: 117 ± 3; day 2: 108 ± 1; day 14: 124 ± 2 mmHg). The detailed post hoc analysis of changes in each day revealed that already on the 14^th^ day the values of BP (both systolic and diastolic) between untreated ACF TGR and ACF TGR treated with sGC stimulator were no different.

### Series 3: Effects of the long-term treatment with sGC stimulator and ACEi on survival the rate, morphometric and histopathological scores (Figs. 6-7, Table 3,  SUPPLEMENTARY FIGURES S1-S5)

The model of ACF-induced HF in TGR is characterized by high mortality rates due to very early onset of decompensation of HF as compared with the course of ACF-induced HF in HanSD rats, as reported in previous studies including ours (Melenovsky et al. 2012; Červenka et al. 2015b; Kratky et al. 2021; Kala et al. 2023).

**Fig. 6:**
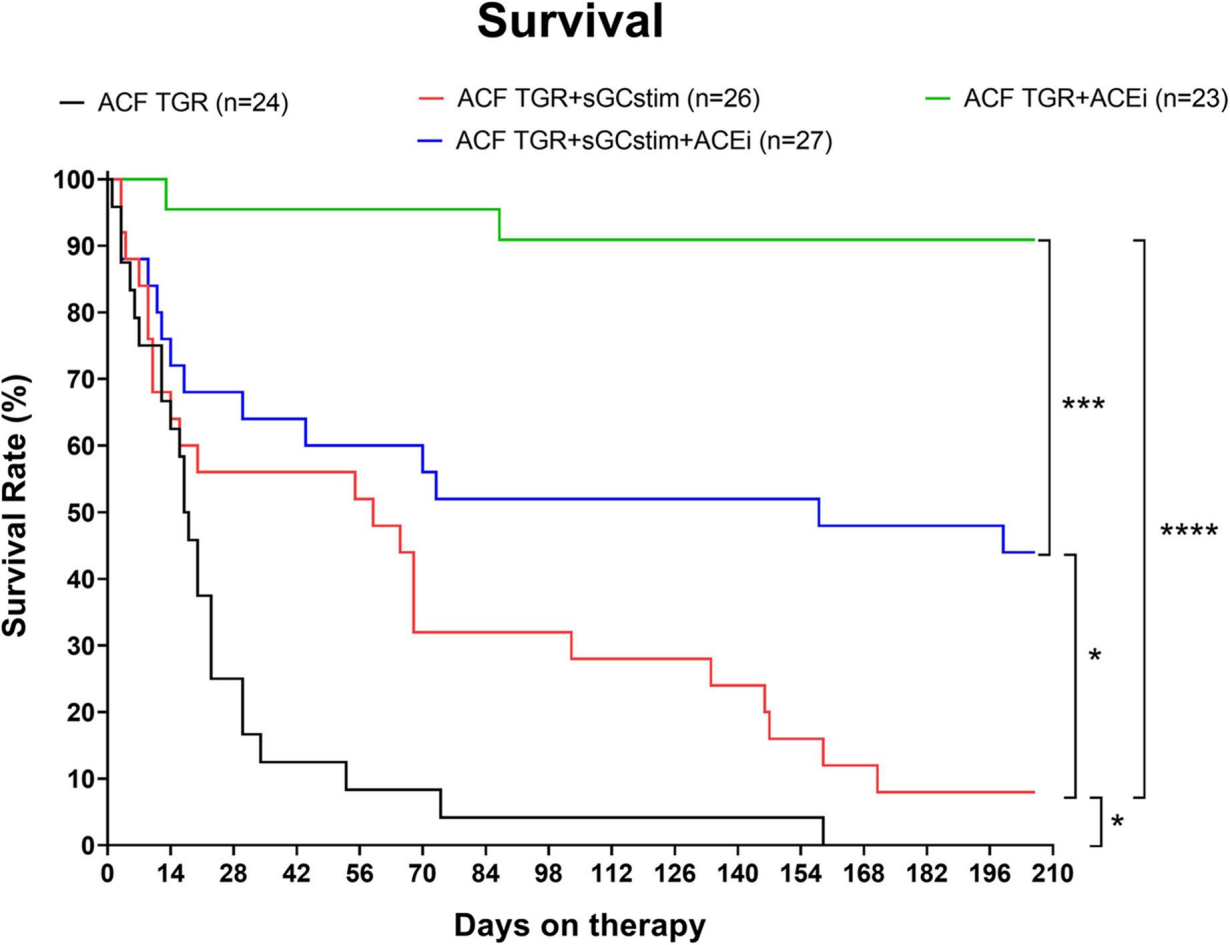
The survival rate in heterozygous Ren-2 transgenic rats (TGR) with aorto-caval fistula (ACF) treated with sGC stimulator (BAY41-8543), or with angiotensin-converting enzyme inhibitor (ACEi), alone or combined. All groups significantly differ between each other (by log-rank Mantel-Cox test); *P* > 0.05 (NS); * *P* ≤ 0.05; ** *P* ≤ 0.01; *** *P* ≤ 0.001; **** *P* ≤ 0.0001; Control normotensive and sham operated TGR groups were omitted for clarity (the survival in these groups was 90–100% in the end)

**Fig. 7:**
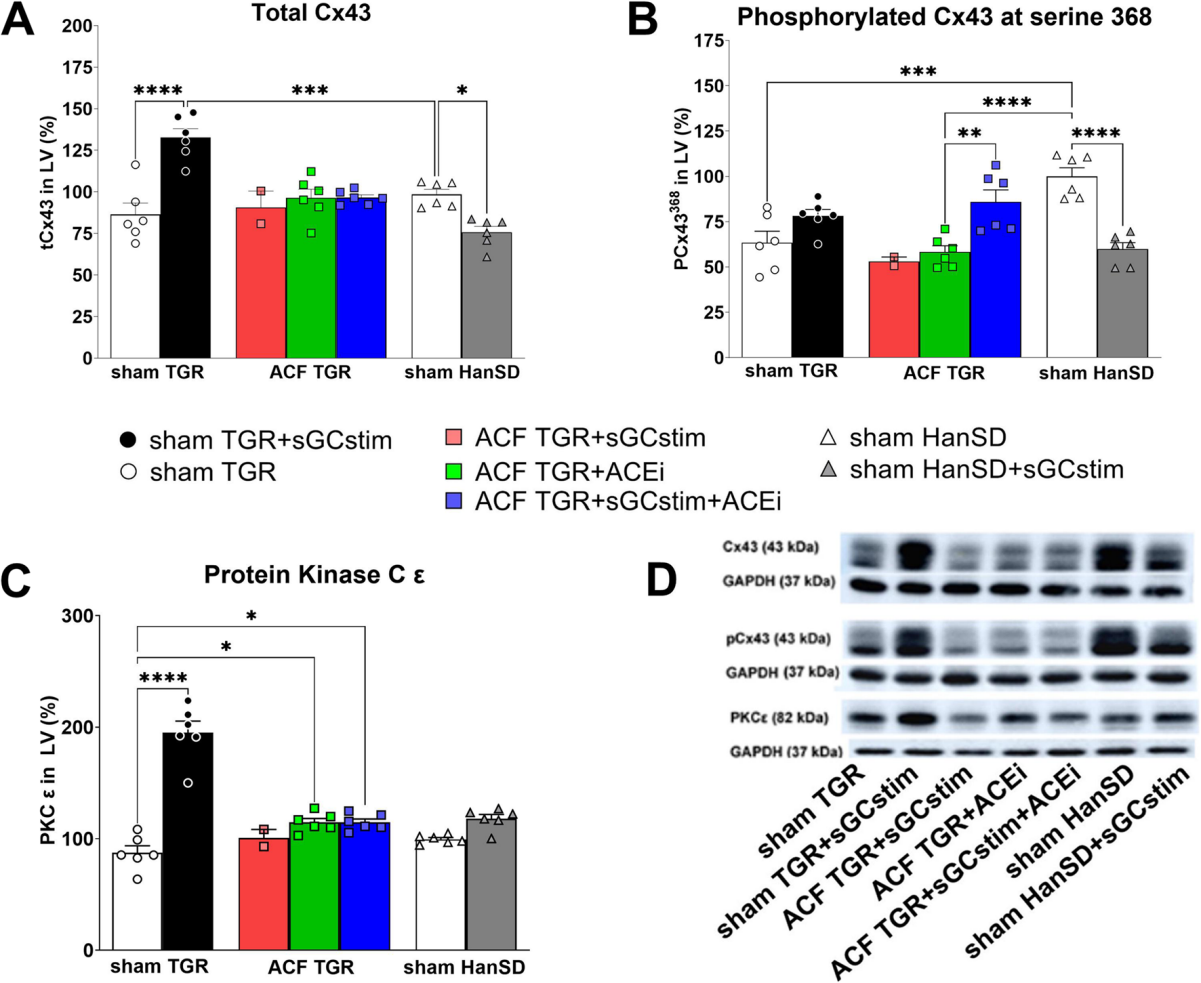
Myocardial protein levels of total Cx43 (**A**), phosphorylated Cx43 at serine 368 (**B**), protein kinase C Epsilon (**C**), and representative western blot images (**D**) measured in left ventricle collected from heterozygous Ren-2 transgenic rats (TGR) rats with aorto-caval fistula (ACF) or without (sham) and normotensive sham HanSD rats that survived until the end of 7 months treatment with sGC stimulator (BAY41-8543), or with angiotensin-converting enzyme inhibitor (ACEi), alone or combined. Values are normalized to GAPDH and presented as mean ± SEM; Cx43 – connexin 43; PKCε – protein kinase C Epsilon; GAPDH- glyceraldehyde-3-phosphate dehydrogenase; *P* > 0.05 (NS); * *P* ≤ 0.05; ** *P* ≤ 0.01; *** *P* ≤ 0.001; **** *P* ≤ 0.0001; by one-way ANOVA and Tukey’s test multiple comparison test; # *versus* all other groups by one-way ANOVA and Tukey’s test multiple comparison test; (*n* = 5 in all groups except ACF TGR + BAY41-8543 where only 2 rats survived; note that no rats survived in untreated ACF TGR group)

**Table 3:** Morphometric and histological parameters measured in material collected from rats that survived until the end of the observation. Heterozygous Ren-2 transgenic rats (TGR) rats with aorto-caval fistula (ACF) or without (sham) and normotensive sham HanSD rats were treated with sGC stimulator (BAY41-8543), or with angiotensin-converting enzyme inhibitor (ACEi), alone or combined for 7 months

	sham TGR (n=8)	sham TGR + sGCstim (n=9)	ACF TGR + sGCstim (n=2)	ACF TGR + ACEi (n=20)	ACF TGR + sGCstim + ACEi (n=12)	sham HanSD (n=9)	sham HanSD + sGCstim (n=10)
	a	b	c	d	e	f	g
BW (g)	765±13 ^f, g^	770±19 ^f, g^	695±43	723±13 ^f, g^	746±25	549±10	592±13 #
Heart W/tibia (mg/mm)	45.6±1.2 ^f, g^	41.7±1.2 ^f,^ *	67.4±0.3	65.6±2.4 ^a, b, f, g^	58.0±3.5^b, f, g^	33.7±0.7	37.1±0.5 #
Lung W /tibia (mg/mm)	46.8±1.3	53.1±2.6	56.0±2.7	57.1±2.1^a, f^	59.1±2.9 ^a, f, g^	44.1±1.2	48.6±0.8 #
Liver W/tibia (g/cm)	5.6±0.2 ^f, g^	5.5±0.2 ^f, g^	4.9±0.6	4.7±0.2 ^b, f^	4.8±0.2 ^f^	3.8±0.1	4.5±0.1 #
Right kidney W/tibia (mg/mm)	49.8±2.1 ^f, g^	47.6±2.0	41.0±8.6	37.5±0.6 ^a, g^	41.9±1.0 ^a, b^	39.1±1.1	44.4±1.7 #
Left kidney W/tibia (mg/mm)	53.1±2.6^f, g^	50.6±2.4	40.0±3.9	37.6±0.8^a, b, g^	42.8±1.7 ^a, b^	39.5±1.0	45.3±2.0 #
GSI	0.43±0.13^f^	0.23±0.01	0.09±0.04	0.10±0.01^a, g^	0.07±0.01^a, g^	0.11±0.02	0.29±0.06 #
TSI	0.26±0.06	0.23±0.06	0.20±0.00	0.06±0.01 ^a, b^	0.08±0.02 ^a, b^	0.13±0.03	0.14±0.02

*BW* – body weight; *W* – weight; *GSI* – glomerulosclerosis index; *TSI* – tubulointerstitial injury;

Superscript letter given next to the value (a-g) indicate statistically significant difference versus group indicated in the second row of the table (under the group’s name) by one way ANOVA with Tukey's multiple comparisons test; * sham TGR + sGCstim vs sham TGR; # sham HanSD + sGCstim vs sham HanSD by unpaired t test. Please note that in ACF TGR+ sGCstim group only 2 rats survived until the end, hence this group was excluded from the statistical analysis, but the values are presented

The survival rates are presented on Fig. 6. All normotensive HanSD rats survived until the end of 210 days observation (100% survival in placebo and in the sGC stimulator treated group); in sham-operated TGR rats (both placebo and sGCstim) the survival was 90% in the end (1 rat died in each group). The sham-operated groups (HanSD and TGR) were omitted for clarity from Fig. 4.

The untreated TGR rats with heart failure (ACF TGR) rapidly started to die in the first few weeks of the observation. This was not attenuated in the initial phase by the sGC stimulator treatment, whereas it was almost stopped from the beginning by the optimized dose of the ACEi. However, in the further progression of the disease phenotype, the stand-alone treatment with sGC stimulator significantly improved survival in comparison to untreated rats (ACF TGR). After 60 days of sGC stimulator treatment the survival was still 50% compared to 8% in the untreated rats. After day 140, the effectiveness of the treatment with the sGC stimulator was then fading out also, however being still significantly better compared to placebo. The best effectiveness on mortality in this experiment was achieved with the optimized ACE inhibitor administered alone (ACF TGR + ACEi); the survival in this group was 90% at the end of the experiment. Surprisingly, the effectiveness of the combined therapy with ACEi and sGC stimulator (ACF TGR + sGCstim) was lower than stand-alone ACEi treatment with 44% at the end of the study.

### Organ weights and renal histopathology scores (Table 3)

At the end of the study (after 210 days), the surviving animals were decapitated and organs were weighted and collected for further analysis. Noteworthy the placebo treated rats with heart failure and the sGC stimulator-treated rats were excluded from the statistical analysis due to the low animal numbers; therefore, this analysis was conducted mainly to compare the effects of the treatments in sham operated animals (both TGR and HanSD) and between ACEi administered alone and combined treatment with ACEi and BAY41-8543.

Out of all ACF rats the lowest heart weight (expressed as a ratio to tibia length) as indicator for heart hypertrophy was in the group treated with the combined treatment with sGCstim and ACEi (58.0 ± 3.5 mg/mm) which was not significantly different from untreated sham TGR (45.6 ± 1.2 mg/mm). Interestingly, the heart hypertrophy in ACF-TGR treated with the combined therapy tended to be lower compared to the ACEi inhibitor treatment alone (58.0 ± 3.5 mg/mm *versus* 65.6 ± 2.4 mg/mm, respectively; *P* = 0.0777 by unpaired T-test; NS). Treatment with sGC stimulator also decreased heart weight in sham TGR (41.7 ± 1.2 mg/mm *versus* 45.6 ± 1.2 mg/mm in untreated animals; *P* < 0.05 by unpaired *T*-test; Table 3).

The lung weight in sham TGR rats treated with sGC stimulator was slightly higher than in untreated sham TGR (53.1 ± 2.6 vs 46.8 ± 1.3, respectively; Table 3), but the difference was not statistically significant. There was no difference in lung weight between applied treatments in ACF TGR groups and all values were lower than in untreated ACF TGR rats at this stage of the disease (due to lack of the data for untreated animals, we can only compare the results to our previous studies). The lung weight to tibia length ratio two weeks after the induction of ACF was 78.79 ± 1.14 mg/mm (Kala et al. 2021) and after four weeks was 74.89 ± 1.43 (Kala et al. 2023), which is significantly higher than in the current study (Table 3).

Except the obvious differences between sham-operated animals *versus* rats with ACF and differences between healthy HanSD rats and hypertensive TGR, there were no major changes in lung, liver and kidney weights between applied treatment regimens. However, in normotensive HanSD rats the treatment with sGC stimulator significantly increased almost all of the measured parameters, when compared to untreated HanSD rats (Table 3, # by *T*-test). Since in this group the increase in body weight (BW) was also the highest we calculated the ratio of organ weights’ to BW which was however, not statistically significant, with the exception of liver weight (sham HanSD + sGCstim: 33.0 ± 0.06 *versus* 30.3 ± 0.05 mg/g in untreated sham HanSD; *P* = 0.003 by unpaired t-Test).

To evaluate the degree of the kidney damage the glomerulosclerosis index (GSI) and kidney cortical tubulointerstitial injury (TSI) were measured (Table 3). The inflammatory cell infiltration, interstitial fibrosis and tubular dilatation were evaluated. Noteworthy all values for GSI and TSI were relatively low, indicating no severe renal damage in all groups (it did not exceed the value of 0.5 in any group, which is considered as a borderline between healthy and renal damage), irrespective of the treatment. However, some subtle changes and significant differences were observed. ACEi administered alone and combined with sGC stimulator decreased GSI (0.10 ± 0.01 and 0.07 ± 0.01, respectively) to the greatest extend and to similar level as in untreated sham HanSD rats (0.11 ± 0.02). Also ACEi (alone and combined with sGC stimulator) decreased TSI when compared to untreated sham TGR and sham TGR treated with sGC stimulator (Table 3). The treatment with sGC stimulator tended to decrease GSI in sham TGR (0.23 ± 0.01 *versus* 0.43 ± 0.13 in untreated sham TGR; NS), but significantly increased it in HanSD rats (0.29 ± 0.06 *versus* 0.11 ± 0.02 in untreated sham HanSD; *P* = 0.017 by unpaired T-test). These changes were not reflected in tubulointerstitial injury score, since TSI was not changed in both sham TGR and sham HanSD (Table 3).

### Protein levels of Cx43 and PKCε assessed by western blot method in left ventricle (Fig. 7)

To evaluate the intercellular communication, the protein levels of Cx43 and PKCε were assessed. Structural remodelling, such as hypertrophy, excessive accumulation of extracellular matrix (ECM) and myocardial fibrosis perturb Cx43 channels mediated intercellular communication in addition to down-regulation of Cx43 due to disease aetiology. The protein levels of the Cx43 phosphorylated at serine 368 characterized by reduced channel conductivity, were significantly supressed in the left ventricle of hypertensive sham TGR and normotensive sham HanSD + sGCstim (63 ± 6 and 60 ± 3 respectively *versus* 100 ± 5% in untreated sham HanSD; *P* < 0.0001 by one way ANOVA).

The protein levels of total Cx43 were significantly decreased in sham HanSD + sGCstim rats (76 ± 4 *versus* 98 ± 3% in untreated sham HanSD; *p* = 0.02 by one way ANOVA). On the contrary, protein abundance of total Cx43 and PKCε, which directly phosphorylate Cx43, was significantly increased in hypertensive sham TGR treated with sGC stimulator (total Cx43: 133 ± 5 *versus* 86 ± 7 and PKCε: 195 ± 10 *versus* 87 ± 6% in untreated sham TGR, respectively; *P* < 0.0001 by one way ANOVA).

### Myocardial topology and quantification of Cx43 (Supplementary FIGURE S1)

In healthy normotensive rats (sham HanSD), Cx43 was detected mainly at the gap junction plaques of the intercalated discs into end-to-end pattern. However, in the hypertensive sham TGR the lateral distribution of Cx43 (side-to-side pattern) was more pronounced. A quantitative image analysis of Cx43 showed a significant decrease of Cx43 in hypertensive sham TGR and normotensive sham HanSD treated with sGC stimulator, compared to normotensive sham HanSD rats.

### Haematoxylin and eosin (HE) histological staining of left ventricle, hydroxyproline assay, and collagen deposition (Supplementary FIGURE S2 and S3)

To evaluate the myocardial remodelling and the level of fibrosis HE staining of left ventricle and hydroxyproline assay was performed. HE staining of left ventricle showed that, compared to sham HanSD rats, the left ventricular tissue of sham TGR and ACF TGR rats exhibited enlarged cardiomyocytes and focal areas infiltrated with polymorphonuclears, mainly in sham TGR (Supplementary FIGURE S2A). Hydroxyproline content (Supplementary FIGURE S2B) in the left ventricular tissue was the highest in untreated sham TGR rats (33.5 ± 2.4 mg/g *versus* 26.9 ± 3.3 in untreated sham HanSD; *p* < 0.05 by one way ANOVA). It seems that all applied treatments diminished the hydroxyproline content, since the values were not different than in healthy normotensive HanSD rats (Supplementary FIGURE S2B). Collagen deposition demonstrated by Van Gieson staining (Supplementary FIGURE S3) showed increased collagen incidence in untreated sham TGR rats compared to healthy normotensive HanSD rats. sGC stimulator treatment slightly normalized this elevated collagen deposition in the left ventricular tissue.

### Myocardial capillary density (Supplementary FIGURE S3 and S4)

Activity of alkaline phosphatase (AP) reflects function and myocardial density of the arterial part of capillaries. AP activity was significantly normalized in sham TGR rats after sGC stimulator treatment (73 ± 6 *versus* 117 ± 11% in untreated sham TGR; *P* < 0.001 by one way ANOVA). No other differences were detected between other groups (Supplementary FIGURE S3). Unlike AP representing the arterial part of capillaries, dipeptidyl peptidase-4 (DPP4) demonstrate a function and density of the venous part of capillaries (Supplementary FIGURE S4). Enhanced DPP4 activity is associated with pathophysiology. Image analysis revealed that the DPP4 activity was significantly increased in heterozygous TGR rats with aorto-caval fistula (ACF) or without it (sham). Treatment did not reveal any significant changes.

## Discussion

The main objective of the present study was to evaluate the effectiveness of sGC stimulator for the treatment of HF in ACF TGR, which is a model of high-output HF associated with development of cardio-renal syndrome. We have shown that BAY41-8543, which exhibits the same mode of action as the sGC stimulator vericiguat, effectively increased the survival of rats with HF with cardio-renal syndrome in comparison to untreated animals.

The discovery of orally active sGC stimulators and sGC activators was a breakthrough in the pharmacology of NO/sGC/cGMP field and seems to have an extensive therapeutic potential. Hence, there is still a great need for pre-clinical and clinical research to fully understand the mode of action and to explore their beneficial activity in cardiovascular and cardio-renal diseases in special patient populations (Cordwin et al. 2021; Sandner et al. 2021b).

In our current study, we employed the rat model of volume overload induced by creation of the aorto-caval fistula, which mimics human HF and t is recommended for pre-clinical research (Riehle and Bauersachs 2019; Kala et al. 2023). The ACF was created in ren-2 transgenic rats, in which the hypertension and endogenous activation of the renin angiotensin system (RAAS) are combined, consequently progressing the development of HF with cardio-renal syndrome (Sobieraj et al. 2021).

The first task in our study was to determine if the selected dose of BAY41-8543 (3 mg kg^−1^ day^−1^) is effective in our model of HF, specifically if it increases the production of secondary messenger cGMP. Therefore, we measured cGMP excretion in urine which was increased after sGC stimulator administration already after one week of treatment. Since the sGC stimulator was administered in the food, we measured the food intake and the concentration of BAY41-8543 in plasma in the end of the short-term experiment. We did not record any differences in the food intake between groups and the plasma levels of sGC stimulator were in accordance to previous studies (Stasch et al. 2015). These data imply that we were able to reach sufficient exposure and target engagement with our sGC stimulator dose. In the next step we verified that our sGC stimulator does not significantly impact blood pressure. Indeed, the treatment with only sGC stimulator proved to have only transient and minor impact on blood pressure in ACF TGR rats.

After having identified the optimal dose, that lead to target engagement but not to significant BP reduction, we conducted a long-term study to evaluate potential beneficial effects of the sGC stimulator BAY 41–8543 in our rat HF model. We have shown that sGC stimulator significantly improved the survival of ACF TGR rats in comparison to untreated animals. This is in line with recent clinical data from the VICTORIA phase 3 pivotal clinical trial which demonstrated that the vericiguat significantly reduced the incidence of the primary outcome of death from cardiovascular causes or first hospitalization for heart failure in comparison to placebo group. The beneficial activity of vericiguat over placebo in the clinical trial appeared after 3 months of treatment and was sustained until the end of 10.8 months follow-up period (Armstrong et al. 2020). In our study the considerable improvement was already visible after three weeks of the treatment (the mortality in untreated rats was already 75% by the 23^rd^ day *versus* only 34% in sGC stimulator-treated group). The beneficial activity of sGC stimulator persisted for another 2 months (by the 59^th^ day of treatment 48% of the rats were still alive), however after that period the effectiveness of the treatment started slowly to fade out. Currently we cannot exclude that this is related to the ACF-procedure and volume overload since preclinical studies in other chronic diseases models have shown long-term prevention (Follmann et al. 2013; Stasch et al. 2015).

Interestingly, we have observed that the optimized dose of the ACEi completely prevented the mortality of the rats, but the efficacy of the ACEi in the combination with the sGC stimulator was substantially reduced. This unanticipated outcome raises some difficulties in interpretation and we cannot provide a plausible explanation. One of the possible hypotheses includes the excessive antihypertensive potency of both drugs in this particular model of HF. As already previously mentioned, the ACF creation causes a significant decrease in blood pressure (around 50 mmHg), therefore our first concept was that the combined treatment causes adverse hypotension, where the values of BP decrease below autoregulatory levels. Low BP is a common symptom in subjects with HFrEF (around 10–15%) and it has been repeatedly demonstrated that blood pressure lower than 90 mmHg is a marker of poor outcome in acute HF (Cautela et al. 2020). Considering the high risk of negative outcomes in patients with HFrEF, current guideline-directed medical therapy recommends the titration of ACEi up to the maximally tolerated dosage (Ouwerkerk et al. 2017; Sharma et al. 2022).sGC stimulators (including BAY41-8543, vericiguat and riociguat) may cause symptomatic hypotension, but it is modest and dose-dependent (Stasch et al. 2002b; Sharkovska et al. 2010; Lam et al. 2021). Moreover, it was shown that even among the population of patients predisposed to hypotension, the efficiency of vericiguat persisted regardless of baseline SBP (Lam et al. 2021). However, we cannot exclude that in this particular model of HF (ACF TGR rats), the effects of the combined treatment (ACEi + sGCstim) depend on the used sGC stimulator dose and/or the sensitivity of this model per se (model specific effect). This is supported by recent findings in ACF TGR model in which the ACEi effects were also blunted by adding an ETA antagonist (Kala et al. 2023).

The clinical importance of NO/sGC/cGMP signalling in cardiovascular and cardio-renal diseases including HF is very obvious; however, a better understanding of the underlying mechanisms is necessary for the optimal HF treatment and prevention and selection of patients profiting best from sGC stimulator therapies (Numata and Takimoto 2022). Hence, the second goal of our study was to further investigate the NO/sGC/cGMP pathway in pathological as well as in physiological states and to further elucidate the possible mechanisms of sGC stimulators. We therefore, have measured a variety of cardiovascular and renal biomarkers and found several interesting findings.

The basal urinary levels of cGMP (before treatment) were significantly higher in all groups in which HF was induced by ACF creation (Fig. 2A). This seems in contrast to data showing impaired cGMP signalling in heart failure (Blanton 2020). However, it seems that NO/sGC/cGMP pathway could be initially activated to counteract the development of HF after ACF creation.

Furthermore, we found that the treatment with sGC stimulator increased or tended to increase the ANG II levels, which also might be in compliance with increased activation of NO/sGC/cGMP signalling pathway mentioned above (both by NO-independent stimulation of sGC stimulator and by endogenous NO). The reciprocal interplay between RAAS and NO/sGC/cGMP is complex and still not fully understood (Persson 2003; Krishnan et al. 2018). cGMP, a secondary messenger in NO/sGC/cGMP cascade, was shown to exhibit both stimulatory and inhibitory action on renin secretion and the results of many studies are contradictory (Kurtz and Wagner 1998; Persson 2003; Curnow et al. 2020). Based on the results of our studies, i.e., increased activity of RAAS after sGC stimulator treatment, we could hypothesise rather stimulatory activity on renin secretion after short term treatment. The increase in cGMP resulting in beneficial effects, such as vasodilation or anti-inflammatory activity, could be counterbalanced by the succeeding activation of RAAS. Noteworthy, this phenomenon was only observed in ACF TGR and sham HanSD rats treated with sGC stimulator, but not in sham TGR, in which the treatment with BAY41-8543 did not affect ANG II levels to such extend (only tendency).

In fact, we have found a number of beneficial effects of sGC stimulator treatment in hypertensive sham TGR group (without ACF creation) exerted both on the renal and cardiovascular systems. The glomerulosclerosis index (GSI) tended to be lower after the sGC stimulator treatment. Furthermore, the analysis of the heart collected after the survival experiment (after 210 days) revealed a great deal of positive activity of sGC stimulator in sham TGR in comparison to untreated hypertensive animals.

Hypertension is linked with an increased occurrence of severe arrhythmias and development and progression of heart failure (Egan Benova et al. 2016). The key factors facilitating such life-threatening events are myocardial structural remodelling, fibrosis and altered topology and disorders of connexin-43 (Cx43) channels. It has been established that down-regulation of Cx43 as well as its abnormal topology contribute to the arrhythmic substrate in failing human heart promoting occurrence of life-threatening arrhythmias, hence the increase in Cx43 appears to be a mechanism to avoid lethal arrhythmia (Danik et al. 2004). We observed a number of positive effects resulted from the long-term treatment with sGC stimulator of hypertensive sham TGR, i.e., decreased heart hypertrophy, significantly up-regulated Cx43 and PKCε in the left ventricle. Moreover, the long term treatment with sGC stimulator tended to decrease hydroxyproline (marker of fibrosis) (Díez 2007) and significantly decreased the activity of alkaline phosphatase (marker of myocardial capillary density) (Schultz-Hector et al. 1993). Certainly, observed beneficial effects of BAY41-8543 treatment can be partially escribed to antihypertensive effectiveness of the sGC stimulator, however the magnitude of the BP decrease, which was only modest suggests also blood-pressure independent beneficial mechanisms.

Taken together, the number of positive effects of the treatment with sGC stimulator in hypertensive TGR rats (without ACF), exerted on the kidney (lower GSI) and heart (diminished hypertrophy, up-regulated Cx43 and PKCε, decreased fibrosis) suggest a great potential of this class of the drug in prevention and/or treatment of persistent hypertension and related disorders.

## Summary and conclusions

The main goal of the current study was to evaluate the effectiveness of sGC stimulator (BAY 41–8543) for the treatment of HF due to volume overload combined with cardio-renal syndrome (ACF TGR). We have shown that the sGC stimulator effectively increased the survival of ACF TGR in comparison to untreated animals. Taken together, we believe that sGC stimulators could represent a valuable tool to treat heart failure and renal dysfunction, but more studies are necessary to elucidate the exact mechanisms of action and interactions with other classes of drugs.

## Limitations of the study

The first limitation of the study is the low numbers of animals, which survived until the end of the survival protocol and the lack of control (untreated) animals for histopathological analysis. The main objective of this part of the study was to evaluate the long-term effectiveness of sGC stimulator and ACEi on the survival of rats with heart failure and cardio-renal syndrome. The initial n numbers of animals used in this study were relatively high (*n* = 30) due to our calculation by statistical power analysis method. During the study, we excluded acute deaths after surgical preparations (ACF creation). Also some of the rats were excluded from the whole analysis due to ACF atrophy, which could be verified only in the end of the observation. Therefore, it was not possible to predict exactly how many samples will be available in the end. Nevertheless, this analysis was conducted mainly to compare the effects of the treatments in sham operated animals (both TGR and HanSD) and between ACEi administered alone and combined treatment with ACEi and BAY41-8543. In the future, we will perform next series of experiments in which the end point will be planned more carefully to avoid this setback.

The second drawback regards the lack of a proper analysis of cardiac function, employing either echocardiography and/or pressure volume analysis. However, that would require repeated usage of anaesthetics, which could impact the results of survival protocol, which was the main goal of this part of the study. Hence, we decided to conduct separate experiments aimed to explore this issue in more detail (in all experimental groups at crucial therapeutic points). This part is still ongoing, due to time-consuming and complex character of the study; therefore, these results will be presented in the future separate studies.

Moreover, we are aware that the analysis of the natriuretic peptide axis is missing. Due to technical reasons, it was not possible in the current study, but it will be included in the next series of experiments in the future.

### Supplementary Information

Below is the link to the electronic supplementary material.

Supplementary file1 (DOCX 3620 KB)

## Data Availability

The datasets generated during and/or analyzed during the current study are available from the corresponding author on reasonable request.

## References

[CR1] Abassi Z, Goltsman I, Karram T, et al (2011) Aortocaval fistula in rat: A unique model of volume-overload congestive heart failure and cardiac hypertrophy. J Biomed Biotechnol 2011:729497 10.1155/2011/729497 10.1155/2011/729497 PMC3025398 21274403

[CR2] Andelova K, Szeiffova Bacova B, Sykora M (2022). Cardiac Cx43 signaling is enhanced and TGF-β1/SMAD2/3 suppressed in response to cold acclimation and modulated by thyroid status in hairless SHRM. Biomedicines.

[CR3] Armstrong PW, Pieske B, Anstrom KJ (2020). Vericiguat in Patients with Heart Failure and Reduced Ejection Fraction. N Engl J Med.

[CR4] Benova T, Viczenczova C, Radosinska J (2013). Melatonin attenuates hypertension-related proarrhythmic myocardial maladaptation of connexin-43 and propensity of the heart to lethalarrhythmias. Can J Physiol Pharmacol.

[CR5] Berliner D, Hänselmann A, Bauersachs J (2020). The treatment of heart failure with reduced ejection fraction. Dtsch Arztebl Int.

[CR6] Blanton RM (2020). cGMP Signaling and Modulation in Heart Failure. J Cardiovasc Pharmacol.

[CR7] Cautela J, Tartiere JM, Cohen-Solal A (2020). Management of low blood pressure in ambulatory heart failure with reduced ejection fraction patients. Eur J Heart Fail.

[CR8] Červenka L, Bíbová J, Husková Z (2015). Combined suppression of the intrarenal and circulating vasoconstrictor Renin-ACE-ANG II axis and augmentation of the vasodilator ACE2-ANG 1-7-Mas axis attenuates the systemic hypertension in Ren-2 transgenic rats exposed to chronic hypoxia. Physiol Res.

[CR9] Červenka L, Melenovský V, Husková Z (2015). Inhibition of soluble epoxide hydrolase counteracts the development of renal dysfunction and progression of congestive heart failure in Ren-2 transgenic hypertensive rats with aorto-caval fistula. Clin Exp Pharmacol Physiol.

[CR10] Ciccarelli M, Dawson D, Falcao-Pires I (2021). Reciprocal organ interactions during heart failure: A position paper from the ESC Working Group on Myocardial Function. Cardiovasc Res.

[CR11] Cordwin DJ, Berei TJ, Pogue KT (2021). The Role of sGC Stimulators and Activators in Heart Failure With Reduced Ejection Fraction. J Cardiovasc Pharmacol Ther.

[CR12] Curnow AC, Gonsalez SR, Gogulamudi VR, et al (2020) Low nitric oxide bioavailability increases renin production in the collecting duct. Front Physiol 11:559341 10.3389/fphys.2020.559341 10.3389/fphys.2020.559341 PMC7705222 33281610

[CR13] Curtis MJ, Alexander S, Cirino G (2018). Experimental design and analysis and their reporting II: updated and simplified guidance for authors and peer reviewers. Br J Pharmacol.

[CR14] Danik SB, Liu F, Zhang J (2004). Modulation of cardiac gap junction expression and arrhythmic susceptibility. Circ Res.

[CR15] Díez J (2007). Mechanisms of cardiac fibrosis in hypertension. J Clin Hypertens (greenwich).

[CR16] Egan Benova T, Szeiffova Bacova B, Viczenczova C, et al (2016) MyocardiaL connexin-43 is implicated in the prevention of malignant arrhythmia in rats suffering from essential hypertension. In: Update on Essential Hypertension. IntechOpen, London

[CR17] Farah C, Michel LYM, Balligand JL (2018). Nitric oxide signalling in cardiovascular health and disease. Nat Rev Cardiol.

[CR18] Follmann M, Griebenow N, Hahn MG (2013). The chemistry and biology of soluble guanylate cyclase stimulators and activators. Angew Chemie - Int Ed.

[CR19] Gawrys O, Baranowska I, Gawarecka K (2018). Innovative lipid-based carriers containing cationic derivatives of polyisoprenoid alcohols augment the antihypertensive effectiveness of candesartan in spontaneously hypertensive rats. Hypertens Res.

[CR20] Gawrys O, Husková Z, Baranowska I (2020). Combined treatment with epoxyeicosatrienoic acid analog and 20-hydroxyeicosatetraenoic acid antagonist provides substantial hypotensive effect in spontaneously hypertensive rats. J Hypertens.

[CR21] Heidenreich PA, Bozkurt B, Aguilar D (2022). 2022 AHA/ACC/HFSA Guideline for the Management of Heart Failure: A Report of the American College of Cardiology/American Heart Association Joint Committee on Clinical Practice Guidelines. Circulation.

[CR22] Honetschlagerová Z, Škaroupková P, Kikerlová S (2021). Effects of renal sympathetic denervation on the course of congestive heart failure combined with chronic kidney disease: Insight from studies with fawn-hooded hypertensive rats with volume overload induced using aorto-caval fistula. Clin Exp Hypertens.

[CR23] Honetschlägerová Z, Hejnová L, Novotný J (2021). Effects of renal denervation on the enhanced renal vascular responsiveness to angiotensin II in high-output heart failure: Angiotensin II receptor binding assessment and functional studies in ren-2 transgenic hypertensive rats. Biomedicines.

[CR24] Husková Z, Kramer HJ, Thumová M (2006). Effects of anesthesia on plasma and kidney ANG II levels in normotensive and ANG II-dependent hypertensive rats. Kidney Blood Press Res.

[CR25] Husková Z, Kramer H, Vaňourková Z (2007). Effects of dietary salt load and salt depletion on the course of hypertension and angiotensin II levels in male and female heterozygous Ren-2 transgenic rats. Kidney Blood Press Res.

[CR26] Husková Z, Kopkan L, Červenková L (2016). Intrarenal alterations of the angiotensin-converting enzyme type 2/angiotensin 1–7 complex of the renin-angiotensin system do not alter the course of malignant hypertension in Cyp1a1-Ren-2 transgenic rats. Clin Exp Pharmacol Physiol.

[CR27] Kala P, Gawrys O, Miklovič M (2023). Endothelin type A receptor blockade attenuates aorto-caval fistula-induced heart failure in rats with angiotensin II-dependent hypertension. J Hypertens.

[CR28] Kala P, Miklovič M, Jíchová Š, et al (2021) Effects of epoxyeicosatrienoic acid-enhancing therapy on the course of congestive heart failure in angiotensin ii-dependent rat hypertension: From mrna analysis towards functional in vivo evaluation. Biomedicines 9(8):1053 10.3390/biomedicines9081053 10.3390/biomedicines9081053 PMC8393645 34440257

[CR29] Kratky V, Kopkan L, Kikerlova S (2018). The role of renal vascular reactivity in the development of renal dysfunction in compensated and decompensated congestive heart failure. Kidney Blood Press Res.

[CR30] Kratky V, Vanourkova Z, Sykora M (2021). AT1 receptor blocker, but not an ACE inhibitor, prevents kidneys from hypoperfusion during congestive heart failure in normotensive and hypertensive rats. Sci Rep.

[CR31] Krishnan SM, Kraehling JR, Eitner F (2018). The impact of the nitric oxide (no)/soluble guanylyl cyclase (sGC) signaling cascade on kidney health and disease: A preclinical perspective. Int J Mol Sci.

[CR32] Kujal P, Čertíková Chábová V, Škaroupková P (2014). Inhibition of soluble epoxide hydrolase is renoprotective in 5/6 nephrectomized Ren-2 transgenic hypertensive rats. Clin Exp Pharmacol Physiol.

[CR33] Kurtz A, Wagner C (1998). Role of nitric oxide in the control of renin secretion. Am J Physiol - Ren Physiol.

[CR34] Lam CSP, Mulder H, Lopatin Y et al (2021) Blood pressure and safety events with vericiguat in the VICTORIA trial. J Am Heart Assoc 10(22):e021094 10.1161/JAHA.121.021094 10.1161/JAHA.121.021094 PMC8751950 34743540

[CR35] Liu R, Kang Y, Chen L (2021). Activation mechanism of human soluble guanylate cyclase by stimulators and activators. Nat Commun.

[CR36] Lojda Z, Gutmann E (1976). Histochemistry of some acid hydrolases in striated muscles of the rat. Histochemistry.

[CR37] McCullough PA, Amin A, Pantalone KM, Ronco C (2022). Cardiorenal Nexus: A Review With Focus on Combined Chronic Heart and Kidney Failure, and Insights From Recent Clinical Trials. J Am Heart Assoc.

[CR38] McDonagh TA, Metra M, Adamo M (2021). 2021 ESC Guidelines for the diagnosis and treatment of acute and chronic heart failure. Eur Heart J.

[CR39] Melenovsky V, Skaroupkova P, Benes J (2012). The course of heart failure development and mortality in rats with volume overload due to aorto-caval fistula. Kidney Blood Press Res.

[CR40] Mullens W, Verbrugge FH, Nijst P, Tang WHW (2017). Renal sodium avidity in heart failure: From pathophysiology to treatment strategies. Eur Heart J.

[CR41] Murphy SP, Ibrahim NE, Januzzi JL (2020). Heart Failure with Reduced Ejection Fraction: A Review. JAMA - J Am Med Assoc.

[CR42] Nakano Y, Hirano T, Uehara K (2008). New rat model induced by anti-glomerular basement membrane antibody shows severe glomerular adhesion in early stage and quickly progresses to end-stage renal failure. Pathol Int.

[CR43] Numata G, Takimoto E (2022). Cyclic GMP and PKG Signaling in Heart Failure. Front Pharmacol.

[CR44] Ouwerkerk W, Voors AA, Anker SD (2017). Determinants and clinical outcome of uptitration of ACE-inhibitors and beta-blockers in patients with heart failure: A prospective European study. Eur Heart J.

[CR45] Pelouch V, Dixon IMC, Sethi R, Dhalla NS (1993). Alteration of collagenous protein profile in congestive heart failure secondary to myocardial infarction. Mol Cell Biochem.

[CR46] Persson PB (2003). Renin: Origin, secretion and synthesis. J Physiol.

[CR47] Rangaswami J, Bhalla V, Blair JEA (2019). Cardiorenal Syndrome: Classification, Pathophysiology, Diagnosis, and Treatment Strategies: A Scientific Statement From the American Heart Association. Circulation.

[CR48] Reddy GK, Enwemeka CS (1996). A simplified method for the analysis of hydroxyproline in biological tissues. Clin Biochem.

[CR49] Riehle C, Bauersachs J (2019). Small animal models of heart failure. Cardiovasc Res.

[CR50] Roberto B, Evora P, M. Evora P, C. Celotto A,  (2012). Cardiovascular Therapeutics Targets on the NO–sGC–cGMP Signaling Pathway: A Critical Overview. Curr Drug Targets.

[CR51] Sandner P, Follmann M, Becker-Pelster E et al (2021a) Soluble GC stimulators and activators: Past, present and future. Br J Pharmacol Oct 2:1–22. 10.1111/bph.15698 10.1111/bph.15698 34600441

[CR52] Sandner P, Zimmer DP, Milne GT et al (2021b) Soluble guanylate cyclase stimulators and activators. In: Schmidt HHHW, Ghezzi P, Cuadrado A (eds) Reactive Oxygen Species. Handbook of Experimental Pharmacology, vol. 264. Springer, Cham, pp 355–394 10.1007/164_2018_197 30689085

[CR53] Schultz-Hector S, Balz K, Bohm M (1993). Cellular localization of endothelial alkaline phosphatase reaction product and enzyme protein in the myocardium. J Histochem Cytochem.

[CR54] Sedláková L, Čertíková Chábová V, Doleželová Š (2017). Renin–angiotensin system blockade alone or combined with ETA receptor blockade: effects on the course of chronic kidney disease in 5/6 nephrectomized Ren-2 transgenic hypertensive rats. Clin Exp Hypertens.

[CR55] Sharkovska Y, Kalk P, Lawrenz B (2010). Nitric oxide-independent stimulation of soluble guanylate cyclase reduces organ damage in experimental low-renin and high-renin models. J Hypertens.

[CR56] Sharma A, Verma S, Bhatt DL (2022). Optimizing Foundational Therapies in Patients With HFrEF: How Do We Translate These Findings Into Clinical Care?. JACC Basic to Transl Sci.

[CR57] Simmonds SJ, Cuijpers I, Heymans S, Jones EAV (2020) Cellular and molecular differences between HFpEF and HFrEF: a step ahead in an improved pathological understanding. Cells 9(1):242. 10.3390/cells9010242 10.3390/cells9010242 PMC7016826 31963679

[CR58] Singh P, Vijayakumar S, Kalogeroupoulos A, Butler J (2018). Multiple Avenues of Modulating the Nitric Oxide Pathway in Heart Failure Clinical Trials. Curr Heart Fail Rep.

[CR59] Sobieraj P, Nilsson PM, Kahan T (2021). Heart Failure Events in a Clinical Trial on Arterial Hypertension: New Insights into the SPRINT Trial. Hypertension.

[CR60] Sporková A, Jíchová S, Husková Z (2014). Different mechanisms of acute versus long-term antihypertensive effects of soluble epoxide hydrolase inhibition: studies in Cyp1a1-Ren-2 transgenic rats. Clin Exp Pharmacol Physiol.

[CR61] Stasch JP, Becker EM, Alonso-Alija C (2001). NO-independent regulatory site on soluble guanylate cyclase. Nature.

[CR62] Stasch JP, Alonso-Alija C, Apeler H (2002). Pharmacological actions of a novel NO-independent guanylyl cyclase stimulator, BAY 41–8543: In vitro studies. Br J Pharmacol.

[CR63] Stasch JP, Dembowsky K, Perzborn E (2002). Cardiovascular actions of a novel NO-independent guanylyl cyclase stimulator, BAY 41–8543: In vivo studies. Br J Pharmacol.

[CR64] Stasch JP, Schlossmann J, Hocher B (2015). Renal effects of soluble guanylate cyclase stimulators and activators: A review of the preclinical evidence. Curr Opin Pharmacol.

[CR65] Sykora M, Kamocsaiova L, Egan Benova T (2019). Alterations in myocardial connexin-43 and matrix metalloproteinase-2 signaling in response to pregnancy and oxygen deprivation of wistar rats: A pilot study. Can J Physiol Pharmacol.

[CR66] Sykora M, Kratky V, Kopkan L, Tribulova N (2023). Anti-Fibrotic Potential of Angiotensin (1–7) in Hemodynamically Overloaded Rat Heart. Int J Mol Sci.

[CR67] Szeiffová Bačova B, Egan Beňová T, Viczenczová C, et al (2016) Cardiac connexin-43 and PKC signaling in rats with altered thyroid status without and with omega-3 fatty acids intake. Physiol Res 65 Suppl 1:S77–90 10.33549/physiolres.933413 10.33549/physiolres.933413 27643942

[CR68] Xia J, Hui N, Tian L (2022). Development of vericiguat: The first soluble guanylate cyclase (sGC) stimulator launched for heart failure with reduced ejection fraction (HFrEF). Biomed Pharmacother.

