# Supplementary material for: The treatment with sGC stimulator improves survival of hypertensive rats in response to volume-overload induced by aorto-caval fistula
Source: Naunyn Schmiedebergs Arch Pharmacol. 2023 Jun 20;396(12):3757–73. doi: 10.1007/s00210-023-02561-y (PMC10643302; doi:10.1007/s00210-023-02561-y)
Supplement: Supplementary file 1 — Supplementary file1 (DOCX 3620 KB) [file 210_2023_2561_MOESM1_ESM.docx]

**SUPPLEMENTARY MATERIAL**

**The treatment with sGC stimulator improves survival of hypertensive rats in response to volume-overload induced by aorto-caval fistula.**

Olga Gawrys^1*^, Zuzana  Husková^1^, Petra  Škaroupková^1^, Zuzana  Honetschlägerová^1^, Zdeňka  Vaňourková^1^, Soňa  Kikerlová^1^, Vojtěch  Melenovský^2^, Barbara  Szeiffová Bačová^3^, Matúš  Sykora^3^, Miloš Táborský ^4^, Luděk  Červenka^1,4^

^1^ Center for Experimental Medicine, Institute for Clinical and Experimental Medicine, Prague, Czech Republic

^2^ Department of Cardiology, Institute for Clinical and Experimental Medicine, Prague, Czech Republic

^3^ Centre of Experimental Medicine, Slovak Academy of Sciences, Institute for Heart Research, Bratislava, Slovakia

^4^ Department of Internal Medicine I, Cardiology, University Hospital Olomouc and Palacký University, Olomouc, Czech Republic

*Corresponding author:

Olga Gawrys, Center for Experimental Medicine, Institute for Clinical and Experimental Medicine, Vídeňská 1958/9, 140 21 Prague, Czech Republic

[olga.gawrys@ikem.cz](mailto:olga.gawrys@ikem.cz); ORCID: 0000-0002-4397-3991


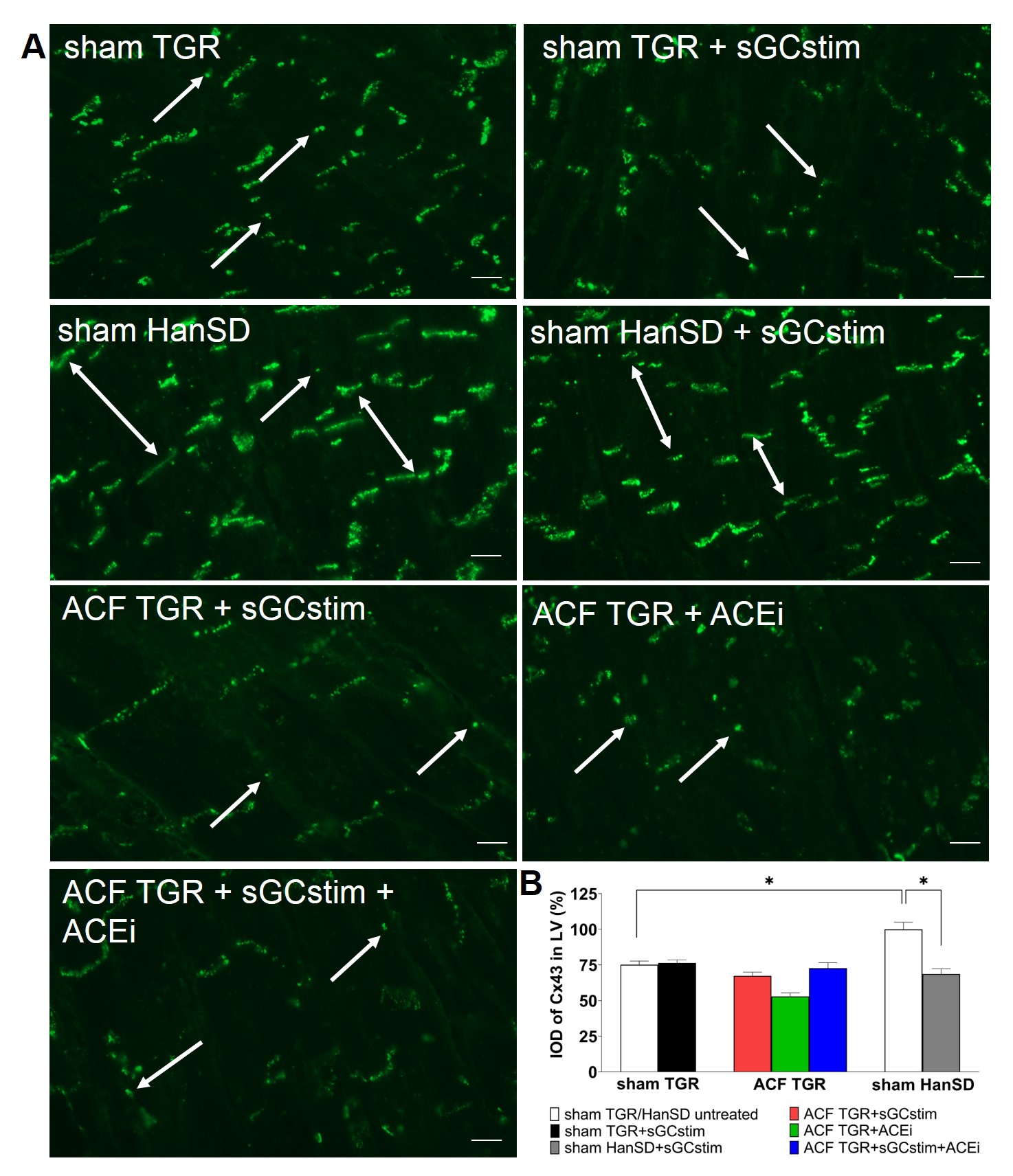


**FIGURE S1:** Immunofluorescence labelling of Cx43 (green) localization (A) and quantitative image analysis of Cx43 (B) represents as total integral optical density per area (IOD) measured in left ventricle collected from heterozygous Ren-2 transgenic rats (TGR) rats with aorto-caval fistula (ACF) or without (sham) and normotensive sham HanSD rats that survived until the end of 7 months treatment with sGC stimulator (BAY41-8543), or with angiotensin-converting enzyme inhibitor (ACEi), alone or combined. Double arrows show end to end pattern of Cx43 and simple arrows lateral pattern of Cx43. Scale bar represents 200 µm. Values are presented as mean ± SEM; * P≤0.05by one-way ANOVA and Tukey's test multiple comparison test (n=5 in all groups except ACF TGR + BAY41-8543 where only 2 rats survived; note that no rats survived in untreated ACF TGR group; approximately 10 randomly acquired images from every tissue were captured and analysed in each group);


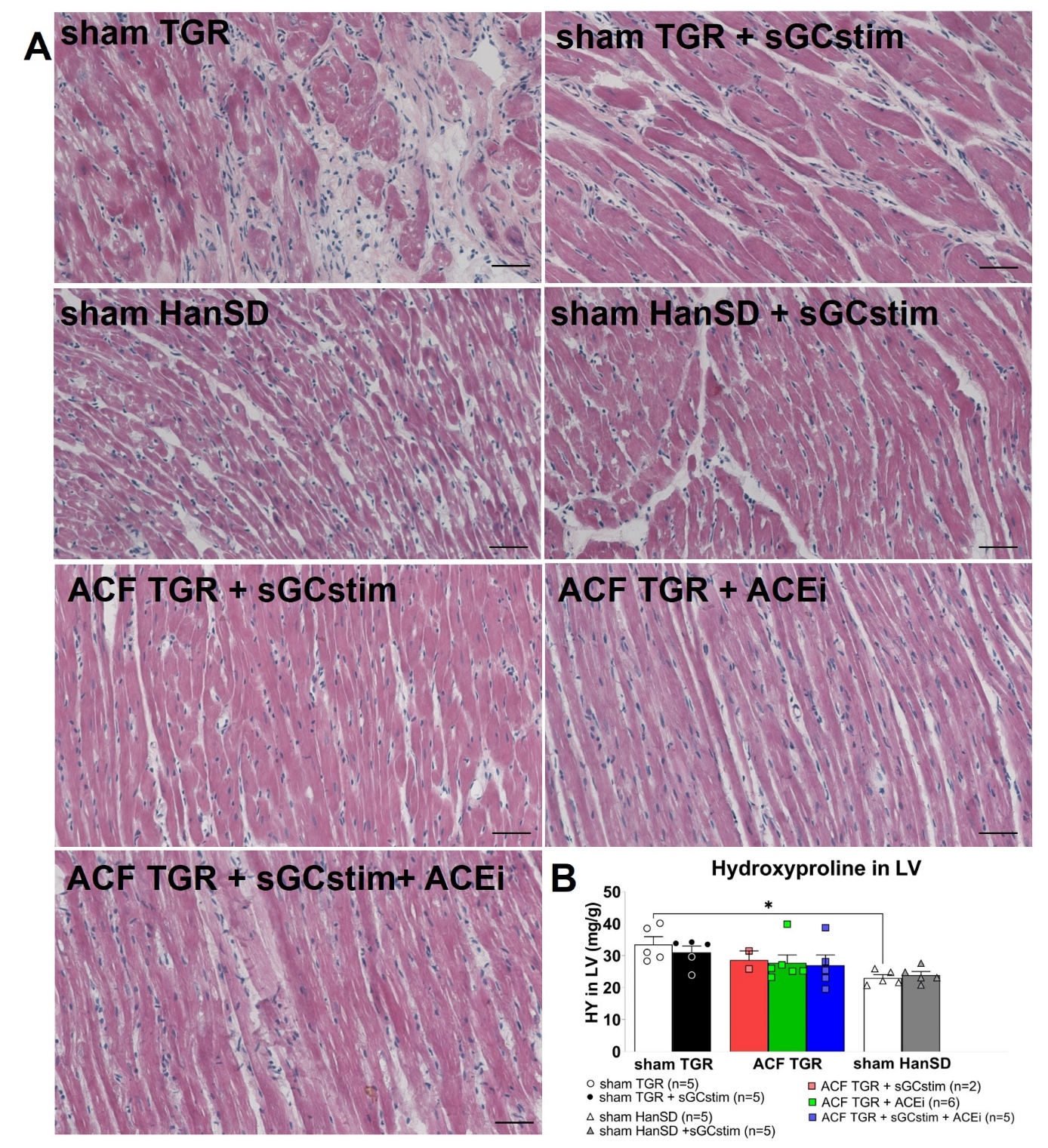


**FIGURE S2:** Hematoxylin–eosin staining (A) and hydroxyproline content detection in the left heart ventricle (B) measured in left ventricle collected from heterozygous Ren-2 transgenic rats (TGR) rats with aorto-caval fistula (ACF) or without (sham) and normotensive sham HanSD rats that survived until the end of 7 months treatment with sGC stimulator (BAY41-8543), or with angiotensin-converting enzyme inhibitor (ACEi), alone or combined. Values are normalized to the weight of tissue used for analysis and presented as mean ± SEM. * P≤0.05 by one-way ANOVA and Tukey's test multiple comparison test (n=5 in all groups except ACF TGR + BAY41-8543 where only 2 rats survived; note that no rats survived in untreated ACF TGR group)


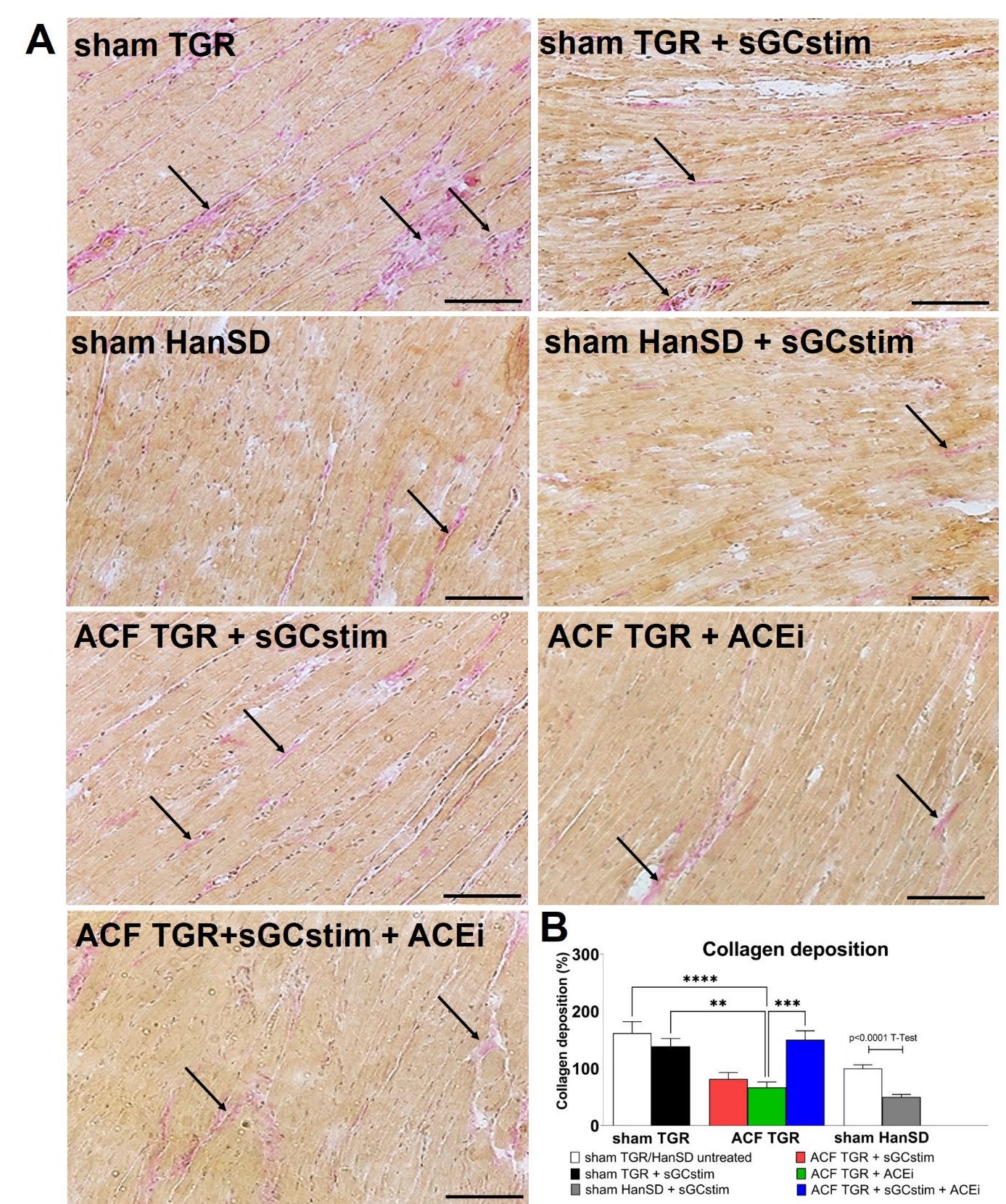


**FIGURE S3:** Histological detection of collagen deposition (pink colour, arrow) by Van Gieson staining (A) and quantification of its evaluation (B) in left ventricle collected from heterozygous Ren-2 transgenic rats (TGR) rats with aorto-caval fistula (ACF) or without (sham) and normotensive sham HanSD rats that survived until the end of 7 months treatment with sGC stimulator (BAY41-8543), or with angiotensin-converting enzyme inhibitor (ACEi), alone or combined. Scale bar represents 100 µm. Values are presented as mean ± SEM * P≤0.05; ** P≤0.01; *** P≤0.001; **** P≤0.0001; by 2way ANOVA with Tukey’s multiple comparisons tests; (n=5 in all groups except ACF TGR + BAY41-8543 where only 2 rats survived; note that no rats survived in untreated ACF TGR group; approximately 10 randomly acquired images from every tissue were captured and analysed in each group);


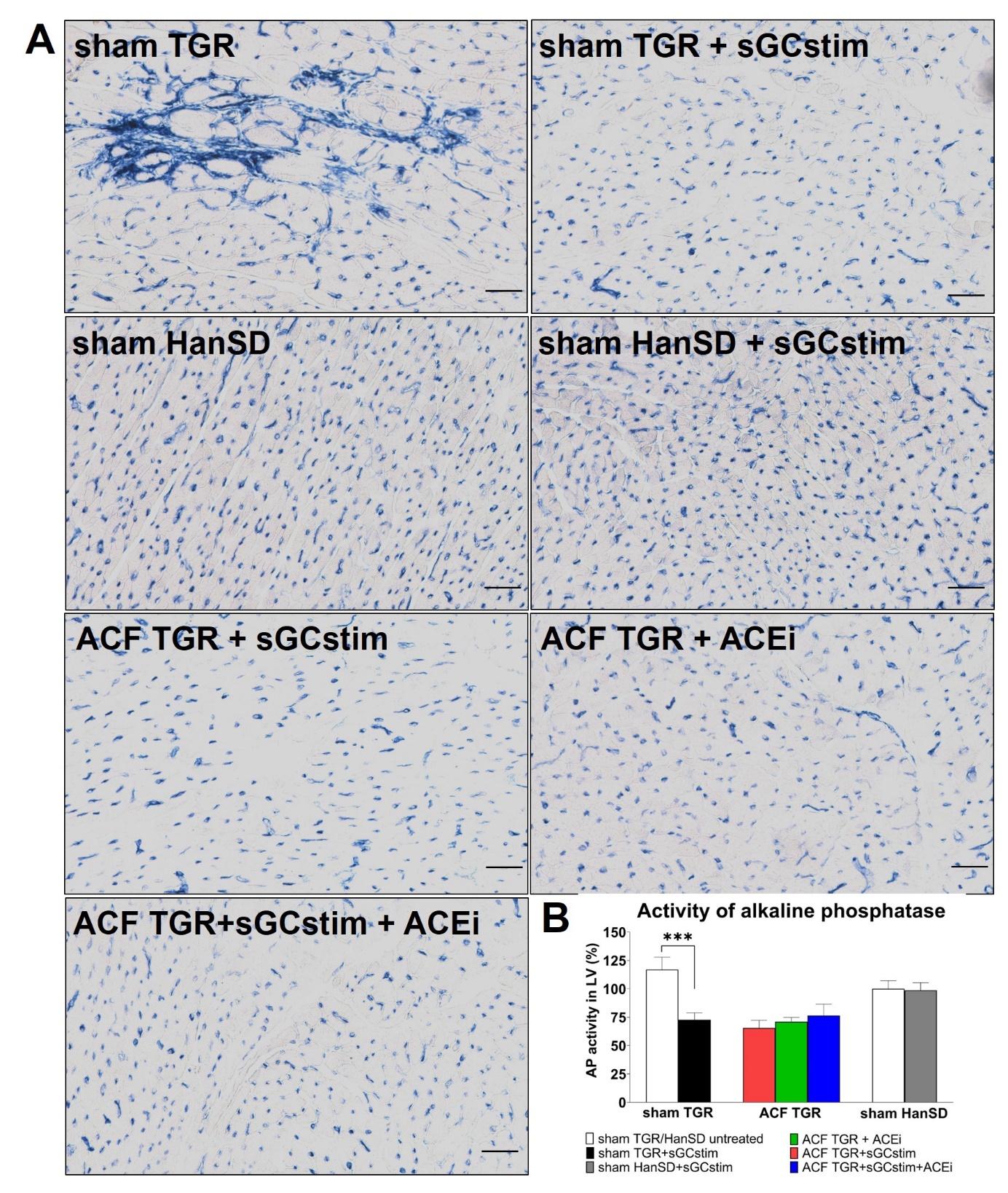


**FIGURE S4:** Myocardial capillary density based on the histochemical demonstration of the alkaline phosphatase (A) activity (blue) and quantification of its evaluation (B) in left ventricle collected from heterozygous Ren-2 transgenic rats (TGR) rats with aorto-caval fistula (ACF) or without (sham) and normotensive sham HanSD rats that survived until the end of 7 months treatment with sGC stimulator (BAY41-8543), or with angiotensin-converting enzyme inhibitor (ACEi), alone or combined. Scale bar represents 50µm. Values are presented as mean ± SEM *** P≤0.001 by one-way ANOVA and Tukey's test multiple comparison test (n=5 in all groups except ACF TGR + BAY41-8543 where only 2 rats survived; note that no rats survived in untreated ACF TGR group; approximately 10 randomly acquired images from every tissue were captured and analysed in each group)


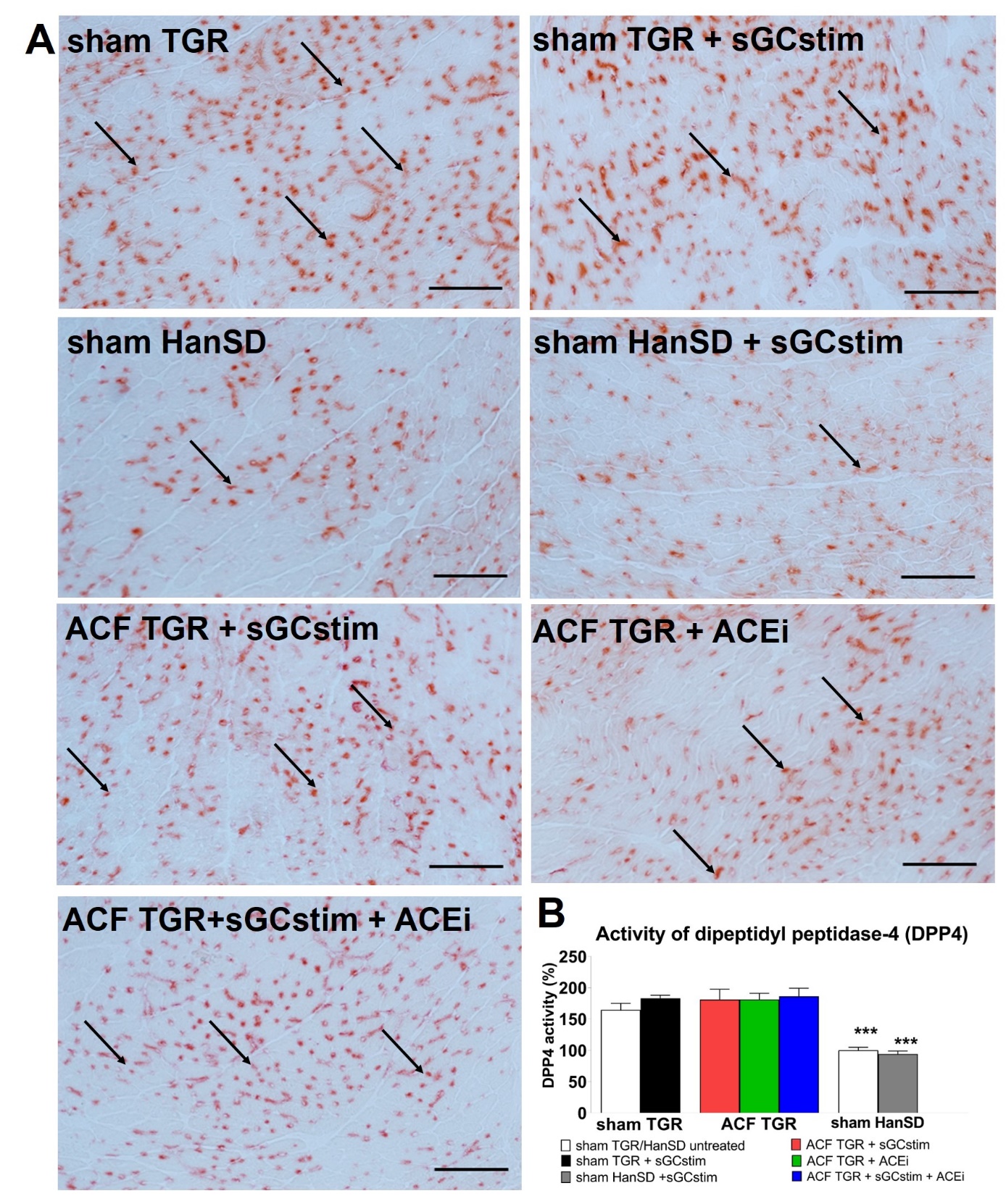


**FIGURE S5**: Activity of dipeptidyl peptidase-4 (DPP4) (red, arrow) in endothelial cells of the venous portion of the capillary network based on histochemical demonstration (A) and quantification of its evaluation (B) in left ventricle collected from heterozygous Ren-2 transgenic rats (TGR) rats with aorto-caval fistula (ACF) or without (sham) and normotensive sham HanSD rats that survived until the end of 7 months treatment with sGC stimulator (BAY41-8543), or with angiotensin-converting enzyme inhibitor (ACEi), alone or combined. Scale bar represents 100 µm. Values are presented as mean ± SEM *** P≤0.001 *versus* all other groups by 2way ANOVA with Tukey’s multiple comparisons tests; (n=5 in all groups except ACF TGR + BAY41-8543 where only 2 rats survived; note that no rats survived in untreated ACF TGR group; approximately 10 randomly acquired images from every tissue were captured and analysed in each group);
